# COVID-19: Is There Evidence for the Use of Herbal Medicines as Adjuvant Symptomatic Therapy?

**DOI:** 10.3389/fphar.2020.581840

**Published:** 2020-09-23

**Authors:** Dâmaris Silveira, Jose Maria Prieto-Garcia, Fabio Boylan, Omar Estrada, Yris Maria Fonseca-Bazzo, Claudia Masrouah Jamal, Pérola Oliveira Magalhães, Edson Oliveira Pereira, Michal Tomczyk, Michael Heinrich

**Affiliations:** ^1^Department of Pharmacy, Faculty of Health Sciences, University of Brasilia, Brasilia, Brazil; ^2^School of Pharmacy and Biomolecular Sciences, Liverpool John Moores University, Liverpool, United Kingdom; ^3^School of Pharmacy and Pharmaceutical Sciences and Trinity Biomedical Sciences Institute, Trinity College Dublin, Dublin, Ireland; ^4^Biophysics and Biochemistry Center, Venezuelan Institute of Scientific Research, Caracas, Venezuela; ^5^Center of Health Sciences, Federal University of Espirito Santo, Vitória, Brazil; ^6^Faculty of Pharmacy, Medical University of Bialystok, Bialystok, Poland; ^7^Pharmacognosy and Phytotherapy, School of Pharmacy, University College of London, London, United Kingdom

**Keywords:** herbal medicine, coronavirus (2019-nCoV), COVID-19, benefit/risk assessment, respiratory diseases

## Abstract

**Background:**

Current recommendations for the self-management of SARS-Cov-2 disease (COVID-19) include self-isolation, rest, hydration, and the use of NSAID in case of high fever only. It is expected that many patients will add other symptomatic/adjuvant treatments, such as herbal medicines.

**Aims:**

To provide a benefits/risks assessment of selected herbal medicines traditionally indicated for “respiratory diseases” within the current frame of the COVID-19 pandemic as an adjuvant treatment.

**Method:**

The plant selection was primarily based on species listed by the WHO and EMA, but some other herbal remedies were considered due to their widespread use in respiratory conditions. Preclinical and clinical data on their efficacy and safety were collected from authoritative sources. The target population were adults with early and mild flu symptoms without underlying conditions. These were evaluated according to a modified PrOACT-URL method with paracetamol, ibuprofen, and codeine as reference drugs. The benefits/risks balance of the treatments was classified as *positive*, *promising*, *negative*, and *unknown*.

**Results:**

A total of 39 herbal medicines were identified as very likely to appeal to the COVID-19 patient. According to our method, the benefits/risks assessment of the herbal medicines was found to be positive in 5 cases (*Althaea officinalis, Commiphora molmol, Glycyrrhiza glabra, Hedera helix*, and *Sambucus nigra*), promising in 12 cases (*Allium sativum*, *Andrographis paniculata*, *Echinacea angustifolia, Echinacea purpurea, Eucalyptus globulus* essential oil*, Justicia pectoralis, Magnolia officinalis*, *Mikania glomerata*, *Pelargonium sidoides*, *Pimpinella anisum*, *Salix* sp, *Zingiber officinale*), and unknown for the rest. On the same grounds, only ibuprofen resulted promising, but we could not find compelling evidence to endorse the use of paracetamol and/or codeine.

**Conclusions:**

Our work suggests that several herbal medicines have safety margins superior to those of reference drugs and enough levels of evidence to start a clinical discussion about their potential use as adjuvants in the treatment of early/mild common flu in otherwise healthy adults within the context of COVID-19. While these herbal medicines will not cure or prevent the flu, they may both improve general patient well-being and offer them an opportunity to personalize the therapeutic approaches.

## Introduction

The outbreak of Coronavirus SARS-Cov-2 disease (COVID-19) in Wuhan (China) in late 2019 and its Worldwide spread has caused hundreds of thousands of deaths so far. As of July 2020, the disease seems to be mostly affecting Europe and the Americas. Most people infected with the COVID-19 virus will experience mild to moderate respiratory illness and recover without requiring special treatment. Older people and those with underlying medical problems such as cardiovascular disease, diabetes, chronic respiratory disease, and cancer are more likely to develop serious illness (WHO, 2020a). Teens and adults without underlying medical conditions are asked to self-manage their symptoms in isolation with a minimum of drugs (paracetamol, if fever is high) and lifestyle adjustments (increased rest and hydration). However, most of the current guidelines do not specifically advise on how to treat cough, one of the main symptoms, which, apart from being very debilitating, contributes to the spread of the virus.

There is not yet any evidence-based specific therapy for COVID-19, and the real efficacy and safety of current therapeutic approaches will need further scrutiny when enough multi-site clinical data become available. The examples of ibuprofen and hydroxychloroquine illustrate how clinical protocols may include and/or exclude drugs in their therapeutic approaches based on limited evidence (Kim et al., 2020; Sodhi and Etminan, 2020; Taccone et al., 2020; Torjesen, 2020). Predictably, patients will largely try to increase their well-being at least by self-administering cough suppressing medication (natural or not) plus natural medication or supplements to combat cold/flu symptoms. These are readily accessible both in retail commerce and community pharmacies. In Europe, there are several herbal medicines registered under the European Directive 24/2004 for self-prescription (EU, 2004). Their labeling establishes that these medicines are indicated for the treatment of common cold and flu symptoms based on traditional use only. We agree in that COVID-19 is not the common flu, but the WHO definition is clear in that it is a mild, self-limiting condition and, therefore, fitting the boundaries of self-prescription, moreover if the patients have not been tested for the virus (WHO, 2020a). In that sense, there is a need to clarify the real potential and safety profile of herbal medicines to scientifically substantiate future recommendations on their benefits and risks of use them. Therefore, the impetus of this work is twofold. First, it intends to highlight which species may provide a more rational phytotherapeutic choice to the disease and second to showcase which plants can be a clinically compatible option as adjuvant therapy for the self-management of common cold/flu symptoms by otherwise healthy adults within the context of the COVID-19 pandemic.

### Clinical Background

Early COVID-19 symptoms include fever, dry cough, and dyspnea, among other similar ones to other viral respiratory diseases such as common flu (Rothan and Byrareddy, 2020). Therefore, the diagnosis of COVID-19 based on anamnesis remains problematic. In general, the incubation period is around 15 days, but the reported range is 0 to 24 days (Bai et al., 2020). SARS-Cov-2 presents a strong transmission capacity (Zheng et al., 2020). To complicate matters further, there is a significant number of asymptomatic patients (up to 60%) who unknowingly contribute to the spread of the disease (Gao et al., 2020; Kronbichler et al., 2020).

The clinical spectrum of the infection goes from mild upper respiratory tract illness to the so-called ‘severe acute respiratory syndrome’: respiratory failure, shock, and multiple organ failure (Bai et al., 2020; Zhou et al., 2020); and may be accompanied by fatigue, headache, diarrhea, and lymphopenia (Rothan and Byrareddy, 2020), and high incidence of cardiovascular symptoms (Zheng et al., 2020). Older people and those with underlying medical problems such as cardiovascular disease, diabetes, chronic respiratory disease, and cancer are more likely to develop severe illness (WHO, 2020a).

The precise pathology of the disease is not yet clear, but seems to include a systemic pro-inflammatory response, inducing hemodynamic changes and, consequently, a predisposition to ischemia and thrombosis (Tang et al., 2020; Zheng et al., 2020; Zhou et al., 2020). A hallmark of the disease is the “cytokine storm”, a massive cytokine and chemokine release due to an uncontrolled dysregulation of the host immune defense that causes loss of function of multiple organs (Catanzaro et al., 2020).

### Preventative and Therapeutic Approaches

Due to the emergence of the propagation of the disease, Health Systems have become overloaded, even having sufficient diagnostic capacity and hospital facilities to handle such an outbreak. In the most vulnerable regions, the COVID-19 epidemic effectively paralyzes health systems at the expense of primary health care (Velavan and Meyer, 2020). Some measures, such as lock-down of communities, social distancing, and quarantine-type for those suspected to be infected can, at least in part, slow the COVID-19 spread (Heymann and Shindo, 2020) and, so, enable the health systems to cope. However, these measures are palliative, and people tend to ignore them after a few days of isolation, mainly those in disadvantaged and vulnerable communities.

Importantly, at this stage, we are starting to build up an evidence-base for the best strategy to treat, mitigate, and prevent the diseases. Currently, none of the approaches used is evidence-based.

In the worldwide search for a response to the COVID-19 pandemic, news about “alternative remedies against COVID” have been disseminated (ANSES, 2020; Nordling, 2020). As of July 2020, the evidence-base for such treatments is often limited if not non-existent. However, often strong, unsubstantiated claims are made about the pros and cons of herbal medicines, which will also result both in false hopes or strong fears of those at risk or ill with COVID-19 (Brennen et al., 2020; Guastalegname and Vallone, 2020; Thorp, 2020).

While some preparations have been claimed to be specifically active, some commonly used medicinal plants are assessed here, especially those on the WHO list of selected medicinal plants, as adjuvant treatments. For example, Hensel et al. (2020), in their recent work, state that extracts prepared with *Echinacea* species (Asteraceae) have an important role with the immune system due to their alkylamide interacting with the cannabinoid receptors (Hensel et al., 2020). Additionally, curcumin, the main constituent in *Curcuma longa* L. (Zingiberaceae), is suggested as a potential clinical option for the treatment of SARS-CoV-2 infection, due to its action in several steps of a viral infection such as protease inhibition, cellular signalling pathways modulation, among others (Zahedipour et al., 2020)

In another paper (Panyod et al., 2020), 11 medicinal plants were discussed concerning their *in vitro* antiviral activity using different models. Two species (*Allium sativum* L. and *Zingiber officinale* Roscoe) mentioned in this study that had their *in vitro* potential and immune capacity assessed, were also included in our study. Moreover, in an elegant evidence-based analysis, eighteen phytotherapeutic preparations were mentioned as having some role in the clinical management of viral respiratory diseases, showing different levels of immunological response (Portella et al., 2020). Four plants mentioned in that report [*Echinacea purpurea* **(**L.) Moench, *Glycyrrhiza glabra* L.*, Sambucus nigra* L., and *Scutellaria baicalensis* Georgi] were also included in our study.

The rationale used in our study was to include, mostly, species known in the Americas and Europe and those already more widely available for the management of respiratory conditions (Blumenthal, 2003; Edwards et al., 2015; Anheyer et al., 2018; Langeder et al., 2020), mainly regarding the symptoms (cough, pain, fever). We recognize that patients suffering from COVID-19 are likely to seek such herbal medications. Complementing other papers published on medicinal plants and their potential to be used for COVID-19, we focused on the therapeutic potential of 39 species, the limitations for their use, and their possible risks. It must be strongly emphasized that this is not an assessment of any mainline treatment for COVID-19 with such herbal medicines. The focus is on assessing their potential as adjuvant therapies to COVID-19. Although the listed herbal drugs included herein have been used for a long time, the evidence level of their action in the relief of mild respiratory symptoms varies, and they are pointed out here.

### The Frame of the Problem

- Apart from a handful of antiviral drugs with limited efficacy, there is only symptomatic therapy for influenza.
- There is no specific therapy for COVID-19.
- There are several herbal medicines recognized by various Health Authorities for the treatment of flu, its symptoms, and other respiratory disorders.
- Health authorities in Europe and the Americas have warned the population against taking any natural/herbal medicines/supplements. They sustain this advice on theoretical potential effects on the immune system due to unspecific anti-inflammatory effects in early stages, facilitating the infection as well as potential immunostimulation, contributing to the aggravation of the “cytokines storm”. As of today, there is no clinical backup for such strong recommendations other than trying to prevent unspecific herb-drug interactions should the patient need emergency care.
- There is a significant OTC use for these herbal medicines, and a realistic prospect is that patients will self-administer them to increase their well-being.

### Aims

To apply a decision-making framework to provide a benefits/risks assessment for selected herbal medicines traditionally indicated for “respiratory diseases” within the current frame of the COVID-19 pandemic.

## Methods

For a treatment to be recommended as adjuvant therapy for respiratory diseases in the context of COVID-19, we here determine that the treatment is effective and that its expected benefits outweigh its potential risks to patients. Briefly, this assessment is informed by the body of evidence about each treatment’s safety and efficacy retrieved in a literature search. This assessment is also informed by a number of other factors, including the severity of the underlying condition and how well patients’ medical needs are addressed by currently available therapies (“reference drugs”). The decision also reflects current applicable laws, regulations, and healthcare recommendations, taking into consideration the uncertainty associated with COVID-19.

### Selection of Herbal Medicines

A search was conducted, considering herbal medicines traditionally used to relieve cold and flu symptoms. The criterion used to limit the investigation, and to grant minimal evidence of efficacy was that the species, linked to the chosen indications, must be listed at least in one of the following organizations: World Health Organization Monographs (WHO); European Medicines Agency (EMA); European Scientific Cooperative on Phytotherapy; ANVISA (Brazilian Pharmacopoea, Brazilian Pharmacopoea Herbal Medicines Formulary, Brazilian Pharmacopoea Herbal Medicines Mementum), Ministry of Health of Chile, Ministry of Health of Cuba, Ministry of Health of Colombia, and Government of Canada. Several South American Countries use the Brazilian Pharmacopeia documents as a reference, so they are also covered within this search. Based on these documents, a list of target species was prepared to establish a search of clinical evidence for the given indication. We refer to these as “herbal medicines” as they are endorsed by Scientific and/or Regulatory committees.

We also included some species which are not listed in monographs based on their significant widespread use in the self-management of respiratory diseases. Some of them are also linked to food uses. We refer to these as “herbal remedies”.

We did not consider species that have given rise to major safety concerns (such as *Ephedra* sp.) and species which are only indicated for relieving mucous phlegm. Furthermore, we do not assess multi-herbal preparations.

### Decision-Making Framework

To assist our decision-making and/or the benefits/risks assessment of herbal medicines, we adopted some of the procedures described in many qualitative or semi-quantitative guidelines to conduct a benefit-risk assessment (PROTECT, 2020). They consist of a step-by-step approach to follow for good decision-making practice and to increase transparency. Descriptive frameworks are usually general, and most of the time, reiterate common sense. Our framework is inspired by the PrOACT-URL method (EMA, 2011c).

The main decision-making elements that we considered for this work are:

- Frame the problem. The appearance of early/mild respiratory symptoms during the pandemic including fever, cough, catarrh, aches, and pains, nasal congestion, runny nose, sore throat, cough, sneezing (= *Condition*) in adults otherwise healthy (= *Target Population*). The patient did not have a test or was negative for COVID-19, but continues at risk of infection (= *Uncertainty*). The patient uses herbal medicines alone or with drugs (= *Treatment*, main or adjuvant). The patient experiences relief of upper respiratory symptoms within 1-2 weeks (= *Favorable effect*). The treatment interferes with hospital/emergency treatment in case of severe acute respiratory syndrome (*Unfavorable effects*).
- Set criteria for Favorable/Unfavorable effects. We followed the “General Guidelines for Methodologies on Research and Evaluation of Traditional Medicine-World Health Organization” for clinical evidence (WHO, 2000) (**Table 1**). We agreed on six key criteria for safety (**Table 2**).
- Consider options to be evaluated against the treatment. Currently available over-the-counter (OTC) medications endorsed by health authorities are non-steroidal anti-inflammatory drugs (NSAIDs) (ibuprofen, naproxen, etc.), antipyretics (acetaminophen/paracetamol), and cough medicines (dextromethorphan, codeine, etc.). They may be taken as monotherapy or alternating therapies. Therefore, one of each category (ibuprofen, paracetamol, and codeine) was chosen to be evaluated based on the same criterion.
- Assess the balance between favorable and unfavorable effects and the associated uncertainty. All possible combinations of clinical and safety grades lead to four possible results: “positive”, “promising”, “negative”, and “unknown”. The last two categories allowed for the inclusion of two different degrees of uncertainty (**Table 3**).
- Recommendation. See conclusions.

**Table 1:** Grading criteria for clinical evidence of the treatments.

Grade	Evidence levels quality/Type of evidence	Requirements
**High**	**Ia** Evidence obtained from meta-analysis of randomized controlled trials **Ib** Evidence obtained from at least one randomized controlled trial	Requires at least one randomized controlled trial as part of the body of literature of overall good consistency addressing the specific recommendation.
**Medium**	**IIa** Evidence obtained from at least one well-designed controlled study without randomization **IIb** Evidence obtained from at least one other type of well-designed quasi-experimental study **III** Evidence obtained from well-designed non-experimental descriptive studies, such as comparative studies, correlation studies, and case-control studies	Requires availability of well-conducted clinical studies with no randomization clinical trials on the topic of recommendation
**Low**	**IV** Evidence obtained from expert committee reports or opinions and/or clinical experience of respected authorities	Requires evidence from expert committee reports or opinions and/or clinical experience of respected authorities. Indicates the absence of directly applicable studies of good quality

Adapted from General Guidelines for Methodologies on Research and Evaluation of Traditional Medicine World Health Organization (WHO, 2000).

**Table 2:** Grading criteria for safety level of the treatments and its evidence.

Grade	Type of adverse effects	Evidence levels quality/Type of evidence*
**High**	A. Other/s than those listed below (B-F). May include allergies, contraindication in pregnancy/lactation, children/elderly, GI disturbances, etc.	**Ia** Evidence obtained from pharmacovigilance data **Ib** Evidence obtained from medically reviewed drug monographs/Patients information leaflets **Ic** Evidence obtained from clinical trials
**Medium**	B. Reported interactions which may affect cardiovascular and/or platelet function C. Presence of compounds potentially able to theoretically produce any in the COVID-19 context (such as coumarins, salicin, ephedrine, etc.) D. No adverse effects reported but known immunostimulant activities	**II** Evidence obtained from case reports or clinical practice/well stablished use. **III** Preclinical evidence in relevant experimental models.
**Low**	E. Reported adverse effects which may affect the respiratory function F. Reported interactions which may affect emergency treatments (anesthesia, mechanical ventilation, etc.)	**IV** Recommendations from expert committee reports or opinions of respected authorities.

*Adapted from General Guidelines for Methodologies on Research and Evaluation of Traditional Medicine World Health Organization (WHO, 2000).

**Table 3:** Benefits/Risks Decision Consensus Criteria.

Clinical evidence (**Table 1**)	Safety evidence (**Table 2**)	Benefits/risks Balance
High	High	Positive
High/Medium	Medium	Promising
Low	High/Medium	Unknown
High/Medium/Low	Low	Negative

### Retrieval of Evidence and Grading

In the second step, we assessed the existing clinical evidence and safety data of all shortlisted herbal medicines and remedies through a bibliographic search, including PubMed, Web Of Science, and other available sources, using the terms “<plant name>” AND cough OR flu OR cold. Also, Cochrane, Drug.com, governmental agencies (EMA, ANSES, and others) were used.

All possible combinations of clinical and safety grades lead to four possible results: “positive”, “promising”, “negative”, and “unknown”. The last two categories allowed for the inclusion of two different degrees of uncertainty. **Table 3** shows how the consensus criteria were translated into a preliminary benefits/risks assessment.

We are aware that for many herbal drug preparations, preclinical evidence exists, and this is not considered in this assessment since it cannot be translated directly into clinical practice. However, such estimation might be of relevance to unveil potential mechanisms of action only so in that case this information was included.

## Results

### Options to Be Evaluated Against the Criteria

Based on the defined parameters, three recommended drugs for early symptoms of COVID-19 - codeine, ibuprofen, and paracetamol - were evaluated.

#### Ibuprofen

Indications in the context of respiratory conditions. Fever, pain, inflammation.

Posology. Up to 2,400 mg a day in doses not bigger than 400 mg every 6 h.

Preclinical evidence. *In vitro* and *in vivo* studies showed evidence of antipyretic and mild analgesic activities (Rainsford, 2015).

Clinical evidence. In a review on the effects of non-steroidal anti-inflammatory drugs (NSAIDs) for treating pain or respiratory symptoms (e.g., cough associated with the common cold), the conclusion was NSAIDs are somewhat effective in relieving the discomfort caused by cold. However, there is no clear evidence of their effect in easing respiratory symptoms. Therefore, the balance of benefits and risks needs to be considered when using NSAIDs for colds (Kim et al., 2013). There is an ongoing clinical trial in the UK with COVID-19 patients receiving a liquid ibuprofen formulation on top of standard care (ClinicalTrials.gov, 2020). Overall, the clinical evidence is High.

Safety. Side effects of ibuprofen include anemia, decreased hemoglobin, eosinophilia, hemorrhage, vomiting, and hypertension. Other side effects include upper gastrointestinal hemorrhage, upper gastrointestinal tract ulcers, dizziness, and dyspepsia. A comprehensive list of very common (10% or more) to common (1% to 10%) adverse effects include nausea (up to 57%), vomiting (up to 22%), flatulence (up to 16%), diarrhea (up to 10%); epigastric pain, heartburn, abdominal distress, indigestion, dyspepsia, abdominal discomfort, constipation, abdominal cramps/pain, fullness of GI tract, bloating, GI hemorrhage, melena (1% to 10%) (Drugs.Com, 2020a). However, regarding COVID-19 patients, there is not enough evidence supporting the potential harmful effects (Sodhi and Etminan, 2020). Overall, safety is Medium.

Specific warnings and precautions of use. Ibuprofen may cause severe cardiovascular thrombotic events, myocardial infarction, and stroke, which can be fatal. This risk may be increased in patients with cardiovascular disease or risk factors for cardiovascular disease. Ibuprofen is contraindicated for the treatment of peri-operative pain in the setting of coronary artery bypass graft (CABG) surgery. NSAIDs can also cause an increased risk of serious gastrointestinal adverse events, especially in the elderly, including bleeding, ulceration, and perforation of the stomach or intestines, which can also be fatal (McGettigan and Henry, 2011).

Overall assessment. According to well-established use, ibuprofen may be useful in the symptomatic relief of respiratory conditions by reducing fever and aches. However, there is only a relatively low number of clinical studies, and meta-analyses do not provide consistent evidence that ibuprofen is effective in reducing symptoms and duration and prevention of the common cold. Overall, the clinical evidence is High. Ibuprofen may have antiplatelet activities; its safety may be considered Medium.

#### Codeine

Indications in the context of respiratory conditions. Cough.

Posology. Up to 360 mg a day in doses not bigger than 60 mg every 4 h.

Preclinical evidence. Some *in vitro, ex vivo*, and *in vivo* studies show evidence of anti-cough activity (Ohi et al., 2007; Cui et al., 2019).

Clinical evidence. Although codeine is widely used as antitussive, the clinical evidence supporting this action is controversial. A study, involving 91 adults presenting cough associated with acute upper respiratory tract infection, showed codeine statistically had the same effect than vehicle (syrup) in controlling cough (Eccles et al., 1992). Overall, the clinical evidence is Low.

Safety. Commonly reported side effects of codeine include drowsiness, lightheadedness, dizziness, sedation, shortness of breath, nausea, vomiting, sweating, and constipation. Other possible effects include bronchospasm, laryngospasm, respiratory depression; heartbeat irregularities, blood pressure changes, syncope, itching, facial swelling, pruritus, urticaria, histamine release; dry mouth, loss of appetite, nausea, vomiting, paralytic ileus, toxic megacolon, anorexia, stomach cramps; miosis, blurred or double vision; euphoria, dysphoria, unusual dreams, hallucinations, insomnia, anxiety (0.1% to 1%); respiratory arrest, dyspnea; flushing, hypotension, palpitations, circulatory depression, shock, cardiac arrest, circulatory depression, bradycardia, tachycardia, edema (Drugs.com, 2019). Overall, safety is Low.

Specific warnings and precautions of use. Death due to respiratory depression has been reported in children (Friedrichsdorf et al., 2013; Tobias et al., 2016). Moreover, codeine can lead to opioid misuse, abuse, and addiction (Casati et al., 2012).

Overall assessment. According to established use, codeine may be useful in the symptomatic relief of cough. However, clinical studies and meta-analyses do not provide consistent evidence that codeine is effective in treating cough. Overall, the clinical evidence is Low. Due to the severe side effects, codeine safety may be considered Low.

#### Paracetamol

Indications in the context of respiratory conditions. Fever, pain.

Posology. Up to 4 g a day in dose not bigger than 1 g every 6 h.

Preclinical evidence. Numerous *in vitro* and *in vivo* studies show evidence of antipyretic and mild analgesic activities (Graham et al., 2013).

Clinical evidence. Paracetamol (acetaminophen) did not show any efficacy in flu, according to a clinical trial involving 80 patients (Jefferies et al., 2016). In a systematic review, the authors concluded that the data did not provide sufficient evidence to inform practice regarding the use of acetaminophen for common cold in adults (Li S. et al., 2013). Overall, the clinical evidence is Low.

Safety. Paracetamol (acetaminophen) is hepatotoxic (Athersuch et al., 2018), and several side effects have been reported (Ishitsuka et al., 2020), such as nausea (up to 34%), vomiting (up to 15%); abdominal pain, diarrhea, constipation, dyspepsia and enlarged abdomen (1% to 10%); anemia, postoperative hemorrhage (1% to 10%); rash, pruritus (1% to 10%); dyspnea, abnormal breath sounds, pulmonary edema, hypoxia, pleural effusion, stridor, wheezing, coughing (1% to 10%); cardiovascular effects (1% to 10%): peripheral edema, hypertension, hypotension, tachycardia, chest pain; metabolic alterations (1% to 10%): hypokalemia, hyperglycemia; headache, dizziness (1% to 10%). Other side effects: dystonia; muscle spasms, trismus (1% to 10%); insomnia, anxiety (1% to 10%); oliguria (1% to 10%); pyrexia, fatigue (1% to 10%) (Drugs.com, 2020b). There are reported cases of deaths in flu patients taking paracetamol (Stevenson et al., 2001). Overall, the safety evidence is Low.

Specific warnings and precautions of use. Not indicated in cases of liver disease and alcoholism (Drugs.com, 2020b).

Overall assessment. According to a well-established use, paracetamol may be useful in the symptomatic relief of respiratory conditions by reducing fever and aches (although a relatively low number of clinical studies and meta-analyses do not provide consistent evidence that paracetamol can reduce symptoms and duration), and prevention of the common cold. The clinical evidence may be considered Low or at best Medium. It is known to be hepatotoxic, and a frequent incidence of respiratory adverse effects may justify serious concerns and a safety rating of Low.

### Assess the Relative Importance of the Decision Maker’s Risk Attitude Towards Herbal Substance

Risk is inherent to any therapeutic intervention (herbal or not). The level of risk of herbal interventions in adults experiencing common flu symptoms without underlying conditions is very low. According to WHO, COVID-19 is self-limiting and mild in this segment of the population (WHO, 2020a). However, we took extra care in integrating current health authorities’ advice with current clinical evidence to emit our assessment.

### Scientific and Clinical Data of Current Herbal Therapy Referred to as Useful to Relieve Symptoms Related to Respiratory Conditions (Cold/Flu)

*General warning:* allergic reactions and gastrointestinal (GI) disturbances are common adverse effects in all medicines and apply to herbal ones. Their use in pregnancy and lactation, babies, children, and the elderly, as well as patients with known severe conditions, is to be individually assessed by a registered healthcare professional.

The recommendations made here are for medicinal products regulated by national authorities to ensure their quality and safety. Other products may be unsafe due to contamination, adulteration, and the presence of naturally occurring toxins in levels above those permitted.

#### *Allium sativum* L. - Amaryllidaceae (Bulbs, Powder)

Indications in the context of respiratory conditions. *Allium sativum* is indicated for respiratory disease, namely cold and cough (CUBA, 2014; EMA, 2017b), and other symptoms related to influenza (BRASIL, 2011). Other related indications of garlic preparations (fresh, garlic powder) included diaphoretic, antiseptic, bacteriostatic, and antiviral effects. It is also used to treat chronic bronchitis and recurrent upper respiratory tract infections (EMA, 2017b; El-Saber Batiha et al., 2020b).

Traditional indications. *Allium sativum* has been traditionally used for alleviation of symptoms of the common cold in adults and children over 12 years. Indeed, garlic is considered as a traditional herbal medicinal product used for the relief of cold symptoms. Moreover, the British Herbal Pharmacopoeia considers that garlic products are indicated for recurrent colds and whooping cough (BHMA, 1983).

Chemical composition. Sulfur compounds (allicin, mercaptan, allyl methyl thiosulphinate, allyl methyl trisulphide, diallyl disulfide, diallyl trisulfide, S-allyl cysteine sulphoxide, and others), glucosides (sativoside B1, proto-degalactotigonin), amino acids (alanine, arginine, aspartic acid, asparagine, histidine, proline, alanine, valine), monoterpenoids (citral, geraniol, alfa and beta-phellandrene and other), peptides, minerals, flavonoids, and vitamins (Lanzotti, 2006; Omar and Al-Wabel, 2010). In the presence of the enzyme alliinase, alliin will be converted to allicin (1 mg alliin to be equivalent to 0.45 mg of allicin). Allicin is also the precursor of other non-volatile products such as ajoenes or oligo- and polysulphides (ESCOP, 2003a; Kovarovič et al., 2019).

Posology (based on traditional uses). Fresh garlic: 2.0–4.0 g average daily dosage (EMA, 2017b). However, it is preferable to use a commercial preparation with defined composition and an adequate dose.

Preclinical evidence. This herbal medicine has been experimentally proven to have antiviral activity. Among the viruses which are sensitive to garlic extracts, are the Human *Cytomegalovirus* (HCMV), *Influenz*a B virus, *Herpes simplex virus* type 1, *Herpes simplex virus* type 2, vesicular stomatitis virus*, Parainfluenza virus* type 3*, Vaccinia virus*, and human *Rhinovirus* type 2 (Tsai et al., 1985; Mikaili et al., 2013). Allicin-containing supplements can prevent attacks by the common cold virus.

Clinical evidence. There is no clinical data to support garlic in the treatment of upper respiratory infections, only in the prevention and treatment of symptoms of the common cold (Josling, 2001). Regarding the prevention or treatment of the common cold, a Cochrane meta-analysis concludes that there is insufficient clinical evidence (Lissiman et al., 2014); the sole study retained for the analysis showed fewer days of illness in the garlic group compared with the placebo group (Josling, 2001). Another trial suggested that consuming the aged garlic extract could reduce the severity of cold symptoms reported (Nantz et al., 2012). Overall, the clinical evidence is High for cold.

Safety. Garlic preparations are generally considered to be safe (EMA, 2016a). However, patients taking anticoagulation and/or antiplatelet therapy should use garlic preparations with caution because they may increase bleeding times (EMA, 2017b). Overall, safety is Medium.

Specific warnings and precautions of use. Patients should avoid concomitant use with anti-platelet drugs.

Overall assessment. Although *Allium sativum* preparations have been used to relieve cold symptoms since ancient times, there is no evidence this herbal medicine can relieve flu symptoms. Whether it may indirectly provide anti-inflammatory and soothing effects on the upper respiratory tract remains to be seen. The clinical evidence may be considered High. Even though garlic is known to have potential antiplatelet effects, overall, it can be considered presenting Medium safety, due to products with less than 0.6% allicin content do not appear to have any such effects (Scharbert et al., 2007).

#### *Althaea officinalis* L. - Malvaceae (Roots, Leaves)

Indications in the context of respiratory conditions. *Althaea officinalis* is indicated for respiratory disease symptoms, namely dry, irritable coughs, and irritations of oral and pharyngeal mucosa (EMA, 2016c).

Chemical composition. Mucilage polysaccharides, such as galacturonorhamnans (rhamnogalacturonan), arabinans, glucans, arabinoglucans, mainly of acidic polysaccharides; flavonoids (e.g., isoscutellarein, hypolaetin, kaempferol and luteolin derivatives); phenolic acids; coumarin (scopoletin); tannins (ESCOP, 2019; Kianitalaei et al., 2019).

Posology (based on traditional uses). 0.5–5.0 g in 150 ml of water as a macerate, three times daily (EMA, 2016c). Marshmallow root’s syrup is also a commonly used preparation in a daily dose of 2.0-8.0 ml (ESCOP, 2019; Kianitalaei et al., 2019).

Preclinical evidence. This herbal medicine has been experimentally proven for respiratory disease symptom, namely, cough. The aqueous extract of marshmallow roots inhibited the tracheobronchial smooth muscle contractions in rats in a dose-dependent manner (Alani et al., 2015). The antitussive effects of oral rhamnogalacturonan (50 mg/kg) were evaluated against mechanically induced cough reflux in both sexes of non-anesthetized cats. The polysaccharide significantly reduced the number of efforts, cough frequency, and intensity of cough attacks from laryngopharyngeal and tracheobronchial areas (Nosalova et al., 2005). The antitussive effects of oral syrup and the polysaccharide were tested against mechanically induced cough of non-anesthetized cats in comparison with non-narcotic antitussives (Nosal’ova et al., 1992).

Clinical evidence. *Althaea officinalis* preparations have been trialed clinically for respiratory disease, and the following symptom was evaluated: cough. In a randomized clinical study, 63 adults suffering from dry cough associated with angiotensin-converting enzyme inhibitors ingested 20 drops, three times per day, of either a marshmallow root preparations or a placebo, for four weeks. The severity of the cough in the marshmallow group was significantly reduced (Rouhi and Ganji, 2007). In one clinical trial on 822 patients with dry cough associated with pharyngeal irritation, the efficacy, tolerability, and satisfaction of *A. officinalis* root aqueous extract in the form of lozenges and syrup was evaluated. *Althaea officinalis* root aqueous extract improved the symptoms of dry cough within 10 min with very good tolerability. There were only three minor adverse events in the syrup group (Fink et al., 2017). Overall, the clinical evidence is High for cough.

Safety. No toxicity was reported at the indicated doses (EMA, 2016b). Overall, safety is High.

Specific warnings and precautions of use. The absorption of other drugs taken simultaneously may be retarded due to the presence of mucilage. As a precautionary measure, all preparations with *A. officinalis* should not be taken 30 min to 1 h before or after intake of other drugs/minerals/vitamins. The macerate should be used immediately after preparation (EMA, 2016b).

Overall assessment. *Althaea officinalis* preparations can suppress cough and diminish irritation through anti-inflammatory and soothing effects on the respiratory tract. Its traditional use as cold therapy in the context of upper respiratory conditions is not backed up by robust clinical data, but the evidence allows us to infer a potential use in the relief of early symptoms of COVID-19. The clinical evidence may be considered High, and as no severe concerns are reported for this herbal medicine, it can be rated as High safety.

#### *Andrographis paniculata (*Burm.f.) Nees - Acanthaceae (Leaves, Aerial Parts)

Indications in the context of respiratory conditions: *Andrographis paniculata* is indicated for respiratory disease, namely common cold, influenza type, and other upper respiratory tract infections, cough, and fever (WHO, 2002). It is often included in multi-herbal preparations, which are not assessed here [aside from the combination with *Eleutherococcus senticosus* (Rupr. & Maxim.) Maxim.].

Chemical composition. Relevant secondary metabolites are diterpenes like andrographic acid, their glucosides (deoxyandrographolide-19β-D-glucoside) and dimers (bis-andrographolides A, B, C, and D) and labdane diterpenoids like andrographolide, neoandrographolide; flavonoids (methoxylated flavones, flavanones, chalcones); steroids (β-sitosterol (Koteswara Rao et al., 2004; Akbar, 2011; Dai et al., 2019; Hanh et al., 2020). Andrographolides are considered the active metabolites and chemical markers of this species.

Posology (based on traditional uses). 1–3 g as a decoction, three times daily (WHO, 2002). However, it is preferable to use a commercial preparation with defined composition and an adequate dose.

Preclinical evidence. This herbal medicine has been experimentally proven for respiratory disease, based on a mouse-model for influenza (an adapted H1N1 strain PR8A/PR/8/34). Treatment with andrographolide decreased the virus loads and the expression of the inflammatory cytokines. Also, diminished lung pathology and overall survival rate (Ding et al., 2017). Anti-inﬂammatory and immunomodulatory properties of the extract of *A. paniculata* and andrographolide have been linked to the increasing proliferation of lymphocytes and the production of IL-2 and inhibition of the tumor cell proliferation immune system (Rajagopal et al., 2003; Kumar et al., 2004). Also, diminished lung pathology and overall survival rate (Ding et al., 2017). Neoandrographolide has *in vivo* anti-inflammatory effects (Panossian et al., 2002). Andrographolide and a standardized registered fixed combination of *A. paniculata* extract SHA-10 and *E. senticosus* extract SHE-3 showed an *in vitro* effect on the activation and proliferation of immune-competent cells as well on the production of key cytokines and immune activation markers (Dai et al., 2019; Kim et al., 2019). A range of anti-inflammatory effects has been reported on diverse disease targets for *A. paniculata* and key constituents (Dai et al., 2019; Kim et al., 2019).

Clinical evidence. This herbal medicine has been trialed clinically for cold symptoms. Three systematic reviews pointed to the beneficial effects and safety of Andrographis for relieving symptoms of acute respiratory tract infections and shortening time to the symptom (Kligler et al., 2006; Akbar, 2011). The most recent study included 33 randomized controlled trials (RCT) with 7175 patients. The results indicated that compared to usual care, a shortening of the duration of symptoms including cough, sore throat, and sick leave/time to resolution was observed. Concerns were raised due to the low quality of many studies and their heterogeneity (Hu et al., 2017; Hu et al., 2018). Overall, the clinical evidence is High for cold and cough.

Safety. An assessment report of EMA concludes that while ‘there is a clear effect on some CYP isoenzymes, the available acute, and reproductive toxicity and genotoxicity data support the safety of Andrographis’ (EMA, 2014a). On the other hand, the Australian Therapeutic Goods Administration (TGA) highlighted the potential risks of severe allergic reactions (TGA, 2015), which, however, seems to be of very limited clinical concern. There are some preclinical indications of immunomodulatory activities of unknown implications for the COVID-19 cytokines storm. Overall, safety is Medium.

Specific warnings and precautions of use. Allergic reactions may be of concern (TGA, 2015).

Overall assessment. Andrographis may be useful in the symptomatic relief of respiratory symptoms, especially in terms of alleviating the symptoms of uncomplicated upper respiratory tract infections. Its traditional use as cold therapy in the context of upper respiratory conditions is not backed up by robust clinical data, but the evidences allow to infer a potential use in the relief of early symptoms of COVID-19. The clinical evidence may be considered High, and as although no severe concerns are reported, this herbal medicine may exert immunomodulatory activities so can be cautiously rated as Medium safety.

#### *Commiphora molmol* Engle [syn. *Commiphora myrrha* (T.Nees) Engl.] and Other *Commiphora* sp. - Burseraceae (Air-Dried Oleo-Gum Resin Exudate)

Indication in the context of respiratory conditions. *Commiphora molmol* is indicated symptoms of respiratory disease, namely mild inflammation of pharyngeal mucosa (WHO, 2007). Other related symptoms are cough, anti-inflammatory (Akbar, 2020); supportive treatment for tonsillitis (WHO, 2007; Barnes et al., 2012; ESCOP, 2014).

Chemical composition. Sesquiterpenes (furanoeudesma1,3-diene and lindestrene as the major components) (Marongiu et al., 2005).

Posology (based on traditional uses). It is preferable to use a commercial preparation with a defined composition, such as a tincture (0.5–5 ml in 150 ml of water for rinsing or gargling three times daily) (WHO, 2007).

Preclinical evidence. This herbal medicine has not been experimentally proven for symptoms of respiratory disease. Although *C. molmol* has been used as a remedy since ancient times (Akbar, 2020), so far, only a few studies on anti-inflammatory or antinociceptive action can be found. An ethanol extract from the resin (at the doses of 100 mg/kg and 200 mg/kg, p.o.) presented analgesic and anti-inflammatory activities in mice. In the swelling paw test, the effect of the extract (100 mg/kg, p.o.) was similar to indomethacin (10 mg/kg, i.p.) (Su et al., 2011).

Clinical evidence. *Commiphora molmol* preparations have not been trialed clinically for respiratory disease, only for inflammation. A standardized extract of *C. molmol* (curzerene 17.93%, furanoeudesma-1,3-diene 27.44%, lindestrene 9.08%) was evaluated about the anti-nociceptive effect. The volunteers (89 men and 95 women), presenting headache, fever-dependent pain, joint pain, muscle aches, lower back pain, or menstrual cramps, received extract (200 mg or 400 mg), or placebo, for 20 days. According to this RCT, the extract presented a similar effect to some frequently used drugs, such as diclofenac, ibuprofen, and paracetamol, although requiring a longer time of use (20 days). In the male group, the extract, at the dose of 400 mg, the effect was significant against all the symptoms. For the female group, the extract was effective against low back pain and fever-dependent pain at the doses of 200 mg/day. Furthermore, no side effect was reported by any of the volunteers (Germano et al., 2017). Overall the clinical evidence is High for pain associated with fever.

Safety. The safety of *C. molmol* is well established. There are no known safety concerns from non-clinical or clinical data (EMA, 2011d; EMA, 2018b). Overall, safety is High.

Specific warnings and precautions of use. Due to a uterine stimulant effect (Vafaei et al., 2020), products containing *C. momol* resin should be avoided in pregnancy and lactation (EMA, 2011d). The concomitant use of *C. molmol* with theophylline or cyclosporine A should be avoided (Al-Jenoobi et al., 2015a; Al-Jenoobi et al., 2015b; EMA, 2018a).

Overall assessment. *Commiphora molmol* preparations seem to have a supportive effect as antinociceptive and thus be useful in the relief of respiratory symptoms. However, its effect is evident only after a few weeks, thus exceeding the normal, uncomplicated evolution of COVID-19. The clinical evidence may be considered High. As no severe concerns are reported, this herbal medicine safety can be rated as High.

#### *Cymbopogon citratus* (DC.) Stapf - Poaceae (Leaves)

Indications in the context of respiratory conditions. *Cymbopogon citratus* is indicated for respiratory infections (CUBA, 2014).

Chemical composition. Essential oil (with geranial, neral and myrcene as the major constituents) (Leclercq et al., 2000; Menut et al., 2000; Pino and Rosado, 2000; Sidibé et al., 2001; Ali et al., 2004; Kanko et al., 2004; Pérez et al., 2006; Rodriguez-Pérez et al., 2006; Brito et al., 2011; Silva et al., 2014; Diop et al., 2017; Soliman et al., 2017; Alam et al., 2018; Silva et al., 2018; BRASIL, 2019a); triterpenes (e.g., cymbopogonol; cymbopogone) (Hanson et al., 1976); flavonoids (e.g., luteolin, apigenin, kaempferol) (Cheel et al., 2005; Orrego et al., 2009; Costa et al., 2015a; Costa et al., 2015b); phenolic acids (e.g., caffeic, chlorogenic, ferulic acids and derivatives) (Tapia et al., 2007).

Posology (based on traditional uses). 1–2 g of dried leaves (or 4–5 g of fresh leaves) in 150 ml, up to three times daily (Carballo, 1995; Matos et al., 2001).

Preclinical evidence. *Cymbopogon citratus* has been experimentally evaluated for fever. However, the evaluation of the antipyretic activity of *C. citratus* herbal tea in rats (p.o. or i.p.) did not result in body temperature reduction (Carlini et al., 1986). On the other hand, other related experimental effects include the anti-inflammatory potential of this species, mainly the essential oil. The essential oil from *C. citratus* has been reported to suppress inhibition of TNF-α-induced neutrophil adherence, inducible nitric oxide synthase (iNOS), and other lipopolysaccharides (LPS)-induced pathways, suppression of COX-2 and peroxisome proliferator-activated receptor alpha (PPAR-α) (Katsukawa et al., 2010; Francisco et al., 2013). Another experiment (murine model of allergic asthma) showed a standardized hexane extract of *C. citratus* led to the reduction of inflammatory cells and eosinophils, as well as the expression of NF-kB/p65, in mice sensitized by Bt-antigen (Machado et al., 2015). Citral, the main substance in *C. citratus* essential oil, presented antinociceptive and anti-inflammatory activity in mice (Quintans-Júnior et al., 2011).

Clinical evidence. This herbal medicine has been not trialed clinically for respiratory disease. Overall, the clinical evidence is Low.

Safety. Some reports considered *C. citratus* safe with no health issues from its usage due to the acceptable concentration limit of the essential oil compounds (Carlini et al., 1986; Ekpenyong et al., 2015). Overall, safety is High.

Specific warnings and precautions of use. None

Overall assessment. This herbal medicine is widely used, and although its profile fit as safety relief therapy for flu, the anti-inflammatory effect on the respiratory tract of *C. citratus* preparations could be useful in the symptomatic relief of respiratory disease. The clinical evidence may be considered Low. As no severe concerns are reported, this herbal medicine safety can be rated as High.

#### *Echinacea* sp. (*E. angustifolia* DC., *E. purpurea* (L.) Moench and *E. pallida* (Nutt.) Nutt. - Asteraceae (Aerial Parts, Rootstock)

Indications in the context of respiratory conditions. This herbal medicine is indicated for symptoms of respiratory disease, namely, those associated with common cold (EMA, 2017c). *Echinacea* preparations are widely used to ‘prevent colds and other respiratory infections, as immunostimulants and in conditions associated with respiratory discomfort. The European Medicine Agency (Herbal Medicinal Product Committee, HMPC) granted registrations for *Echinacea purpurea* (L.) Moench (purple coneflower) for a preventive continuous use for maximal ten days, specifically excluding pediatric populations and with autoimmune illnesses as a contraindication (EMA, 2017a).

Chemical composition. Alkylamides, polysaccharides. Caffeic acid derivatives serve as marker substances (e.g., echinacoside for *E. pallida* root; cichoric acid for *E. purpurea* aerial parts) but are not considered to be of therapeutic relevance (Edwards et al., 2015).

Posology. It is essential to use a commercial preparation with defined composition and an adequate dose. Preparations that administer the extract to the upper respiratory tract (like lozenges) may well be preferable over solid formulations.

Preclinical evidence. This herbal medicine has been not experimentally proven for respiratory disease, namely cough, fever, flu, cold (EMA, 2017a). *In vitro* studies were reported against a range of respiratory viruses but for some preparations only at higher concentrations (Pleschka et al., 2009; Hudson and Vimalanathan, 2011). The immunomodulatory effects of a standardized extract of *Echinacea purpurea*, as well as fractions rich in chicoric acid, polysaccharide, and alkylamide, were evaluated in rats. Phagocytic activity of alveolar macrophage was increased with increasing concentrations of the Echinacea components. Also, a trend of increase in TNF-α and NO release, after *in vivo* LPS stimulation, by the alveolar macrophages was observed. High concentration led to a release of cytokines (such as TNF-α and IFN-γ) in rat’s spleen macrophage (Goel et al., 2002).

Clinical evidence. Echinacea preparations have been trialed clinically for flu and cold. However, the overall problem with the assessment of the evidence refers to the variability of the pharmaceutical preparations investigated in clinical studies (Linde et al., 2006; Karsch‐Völk et al., 2014; David and Cunningham, 2019). Therefore, a large number of clinical studies, as well as a range of pharmacodynamics studies, have been conducted, overall indicating at best a weak evidence for benefits for treating colds. Several metanalyses failed to find any evidence of clear benefits. Still, two RCT reported statistically significant benefits for patients in terms treated with *E. purpurea* and *E. angustifolia* preparations of both symptoms and reduced duration of flu or cold (Linde et al., 2006; Karsch‐Völk et al., 2014; David and Cunningham, 2019), thus qualifying for A (Ib). Of note, one study showed no difference between an *Echinacea pallida* preparation and oseltamivir (Rauš et al., 2015). Overall, the clinical evidence is High for cold and flu.

Safety. Echinacea preparations are generally considered to be safe, although some allergic reactions have been recorded (EMA, 2017a). Interactions risks seem to be of no therapeutic concern (Modarai et al., 2010). Although French Agency (ANSES) postulated that echinacea, among other herbal medicines, could interfere with the immune response in the context of COVID-19 pandemic (ANSES, 2020), at this stage, there is no evidence to support this and, more generally, herbal treatments are not known to rigorously block inflammatory processes and to negatively affect immune responses. Therefore, these concerns seem to be a theoretical postulate which would require further evaluation. While there are no clinical signals for interaction with other medications, ANSES’s concerns might be linked to theoretical interaction with immunosuppressants (such as ciclosporin and methotrexate) due to an antagonistic effect (Williamson et al., 2013). Overall, safety is Medium.

Specific warnings and precautions of use. Like all other products discussed here, there is no evidence for specific therapeutic benefits, and it is important to communicate this to potential users. The safety concerns listed above need to be kept in mind.

Overall assessment: *Echinacea* sp. may be useful in the relief of respiratory symptoms by exerting a soothing effect on the respiratory tract. Overall, a relatively large number of clinical studies and a series of meta-analyses provide evidence that *Echinacea* preparations seem to be efficacious both in the treatment (reducing symptoms and duration) and prevention of the common cold. The clinical evidence may be considered High except for *E. pallida*. Although in the COVID-19 context, caution needs to be taken in order to avoid immunostimulation in complications in later phases of the disease, this herbal medicine safety can be rated as Medium.

#### *Eucalyptus globulus* Labill. - Myrtaceae (Leaves, Essential Oil)

Indication in the context of respiratory conditions. *Eucalyptus globulus* is indicated for symptoms of respiratory disease, namely cough (WHO, 2002; BRASIL, 2011; EMA, 2013a). Other related indications include respiratory antiseptic (CHILE, 2010) and expectorant (COLOMBIA, 2008), due to the presence of 1-8-cineol (Fischer and Dethlefsen, 2013; Salehi et al., 2019b).

Chemical composition. Essential oil (1,8-cineol as the major component); phenolic acids (cafeic, ferulic acid, and derivatives), tannins (gallic and protocatechuic acids), flavonoids (quercetin derivatives) (Dixit et al., 2012; Sonker et al., 2017).

Posology (based on traditional uses). 1.5–3 g of dried leaves in 150 ml, up to four times daily (EMA, 2013a).

Preclinical evidence. This herbal medicine has not been experimentally proven for symptoms of respiratory disease. *In vitro* and *in vivo* studies with leaves extracts, essential oil, and 1,8-cineol are supportive for some ethnomedicinal use (Ross, 2001; Jun et al., 2013; Brezáni et al., 2018; Dhakad et al., 2018; Galan et al., 2020); for example, the essential oil (300 mg/kg) exerted an anti-inflammatory effect on LPS-induced bronchitis in rats, inhibiting the airway mucin hypersecretion (Lu et al., 2004).

Clinical evidence. *Eucalyptus globulus* essential oil preparations have been trialed clinically for respiratory disease (bronchitis, rhinitis), and the following symptoms were evaluated: cough and throat irritation. In an aromatherapy experiment with 48 students diagnosed with allergic rhinitis, eucalyptus reduced coughing, itching sensation in the throat and oral cavity, as well as other symptoms. After four weeks of treatment, the volunteers related a reduction of the discomfort provoked by rhinitis (Song and Kim, 2014). An RCT, involving 152 volunteers with acute non-purulent rhinosinusitis, was carried out with 1,8-cineol, the main compound of eucalyptus essential oil (capsules of 200 mg of oil or placebo, three times daily). After four and seven days, the cineol group presented better symptoms scores than the control group. Moreover, inflammatory processes, such as bronchitis, pharyngitis, tracheitis, conjunctivitis, were less frequent among the verum group (Kehrl et al., 2004). Overall, the clinical evidence is Medium for bronchitis and cough for the essential oil, while for the herbal drug is Low.

Safety. In traditional doses, there is no report on the toxicity of *E. globulus*. However, high doses can cause nausea, vomiting, and diarrhea (WHO, 2002). In a preclinical assay of 1,8-cineol in mice, this compound was classified as presenting low toxicity (Xu et al., 2014). However, high doses can cause nausea, vomiting, and diarrhea (WHO, 2002). However, in the cited clinical trial, the essential oil and 1,8-cineol did not present a significant side effect. Overall, safety is High.

Specific warnings and precautions of use. The use is contraindicated for patients with hypersensitivity to the active substance. Moreover, this plant should not be used by children under 30 months of age due to 1,8-cineole containing preparations, which can induce laryngospasm (EMA, 2013a). In rats, 1,8-cineol induced CYP-450 activity and reduced the levels of amphetamine, pentobarbital, and aminopyrine in plasma or brain (Jori et al., 1970). Moreover, the usual precautions relevant to essential oils should be taken into account.

Overall assessment. *Eucalyptus globulus* may be useful in the relief of symptoms associated with upper respiratory infection by exerting a soothing effect on the respiratory tract. However, even though the extensive use of products containing eucalyptus derivatives, more evidence is need on the impact in the respiratory tract. The clinical evidence may be considered Medium. Although there are concerns about the eucalyptus use by babies, this herbal medicine safety can be rated as High.

#### *Foeniculum vulgare* Mill. – Apiaceae (Fruits)

Indications in the context of respiratory conditions. *Foeniculum vulgare* is indicated for respiratory disease, namely cough associated with cold and fever (EMA, 2007; WHO, 2007).

Chemical composition. Essential oil (trans-anethole, estragole, and limonene as major components) (Singh et al., 2006; Badgujar et al., 2014); stilbenes (e.g., foeniculosides X and XI, cis- and trans-miyabenol) (De Marino et al., 2007); flavonoids (e.g., eriodictyol, quercetin, and derivatives) (Parejo et al., 2004; Faudale et al., 2008); and triterpenes and steroids (e.g., oleanolic acid, 7α-hydroxycampsterol) (Parejo et al., 2004; De Marino et al., 2007; Rather et al., 2016).

Posology. 1.5 to 2.5 g in 200 ml of boiling water (brew for 15 min) three times daily (EMA, 2007; WHO, 2007).

Preclinical evidence. This herbal medicine has been experimentally proven for cough. The ethanol extract, as well as the essential oil of *F. vulgare* presented analgesic and anti-inflammatory activity in rats (Tanira et al., 1996; Özbek, 2005; Him et al., 2008; Araujo et al., 2013; Elizabeth et al., 2014). The aqueous and ethanolic extract and essential oil of *F. vulgare* were evaluated about the myorelaxant activity using isolated guinea-pig trachea, as a model to bronchodilatory effect. The ethanolic extract and essential oil exert relaxant effects similar to those presented by theophylline. The aqueous extract, on the other hand, presented a contraction effect. The myorelaxant effect was not due to inhibition of muscarinic and histamine H1 and/or stimulation on β2-adrenergic receptors (Boskabady and Khatami, 2003).

Clinical evidence. *Foeniculum vulgare* has not been trialed clinically for respiratory diseases. Overall, the clinical evidence is Low.

Safety. Although essential oil of *F. vulgare* presents estragole as one of the main components, and due to its genotoxic carcinogenicity, the exposure to this compound should be kept as low as possible (EMA, 2019), in the recommended herbal preparation and posology, *F. vulgare* is considered safe. Overall, safety is High.

Specific warnings and precautions of use. The safety of fennel was long considered to be of no concern and importantly linked to the long history of use as a medicine and food. The German Commission E lists no risks. In recent years, concerns were raised related to the content of estragole (methyl chavicol), which is known as a potential carcinogen. No clinical reports of fennel’s toxicity are known. The dose and the relevance of some studies with pure estragole at high doses have been disputed. Overall, the evidence is very limited, and there is no known reason for concern (Edwards et al., 2015). Administration of different doses of fennel essential oil reduced the intensity of oxytocin and PGE2 induced contractions significantly (25 and 50 µg/ml for oxytocin and 10 and 20 µg/ml for PGE2, respectively) (Ostad et al., 2001). Fennel is a CYP3A4 inhibitor and can interfere in the metabolism of several drugs (Subehan et al., 2006).

Overall assessment. Even though its profile fits as safety relief therapy for cough in the context of upper respiratory affections, therapeutic benefits are likely to be limited. The clinical evidence is Low. This herbal medicine safety can be rated as High, although the recommended dosage must be observed.

#### *Glycyrrhiza glabra* L. - Fabaceae (Roots)

Indication in the context of respiratory conditions. *Glycyrrhiza glabra* is indicated for symptoms of respiratory disease, namely, cough, sore throat (WHO, 1999; EMA, 2012a).

Chemical composition. Saponins (e.g., glycyrrhizin); triterpenes (glycyrrhetinic acid); flavonoids (liquiritin, rhamnoliquirilin, liquiritigenin, besides others); coumarins (e.g., licoarylcoumarin); essential oil (Saxena, 2005; Öztürk et al., 2017; Frattaruolo et al., 2019; El-Saber Batiha et al., 2020a).

Posology (based on traditional uses). 1.5 g of roots in 150 ml, as herbal decoction two times daily (EMA, 2012a).

Preclinical evidence. This herbal medicine has not been experimentally proven for cold symptoms. Other related experimental effects are anti-asthma and antiviral. The anti-asthma activity of licorice was proven in sensitized rats. A crude hydroethanolic extract (100 mg/kg, p.o) exerted a similar effect to prednisolone (10 mg/kg, p.o.) in mast cells degranulation (Patel et al., 2017). Glycyrrhizin improved the survival time of mice infected with the *Influenza* virus. Also, inhibited the SARS-related coronavirus proliferation *in vitro*. Glycyrrhizic acid inhibited the growth of the *Influenza* virus, inflammatory cytokines, as well as the cytopathic effect of the Respiratory Syncytial Virus (RSV) (Fiore et al., 2008).

Clinical evidence. *Glycyrrhiza glabra* preparations have been trialed clinically for respiratory disease (asthma). An RTC, involving 36 patients presenting chronic asthma, showed licorice in a dose of 3.5 mg/kg in 200 ml water, three times daily, was able to improve the pulmonary function’s parameter similarly to prednisolone (0.15 mg/kg) as a single daily dose (Al-Jawad et al., 2012). Overall, the clinical evidence is High for asthma.

Safety. Patients affected by hypertension, kidney diseases, liver or cardiovascular disorders, or hypokalemia, should avoid *G. glabra* (Nazari et al., 2017). On the other hand, licorice is widely used in food preparation and, therefore, short term uses seem of little concern in otherwise patients with no history of major diseases. Overall, safety is High.

Specific warnings and precautions of use. In an *in vitro* experiment, licorice ethanol extract inhibits CYP3A4 and CYP2D6 (Budzinski et al., 2000; Pandit et al., 2011).

Overall assessment. *Glycyrrhiza glabra* is used for a long time and can be useful in the relief of respiratory symptoms by exerting a soothing effect on the respiratory tract. The clinical evidence can be considered as High. This herbal medicine safety can be rated as High, although it should be avoided for some risk groups.

#### *Hedera helix* L. - Araliaceae (Leaves)

Indication in the context of respiratory conditions. *Hedera helix* is indicated for some symptoms of respiratory disease, namely expectorant (a medicine that helps to bring up phlegm) for productive (chesty) coughs (EMA, 2015a). Other related indications include its actions as antispasmodic and in the treatment of flu and fever (Bisset, 1994; Blumenthal et al., 1998; Hong et al., 2015; Kruttschnitt et al., 2019).

Chemical composition. Flavonoids and other phenolics (Urban, 1958; Trute and Nahrstedt, 1997; Al-Snafi, 2018); polyacetylenes (falcarinol, dehydrofalcarinol) (Bohlmann et al., 1961; Gafner et al., 1989); saponins (Elias et al., 1991; Crespin et al., 1995; Yakovishin and Grishkovets, 2018); and essential oils (β-caryophyllene, germacrene D, limonene, α- and β-pinene, and sabinene as the main components) (Tucker and Maciarello, 1994).

Posology. Pharmaceutical preparation with a defined chemical profile and an adequate dose need to be used (EMA, 2015a).

Preclinical evidence. This herbal medicine has been experimentally proven for symptoms of respiratory disease, namely productive cough (Hong et al., 2015; Pizzorno et al., 2016). Other related experimental effects include acute and chronic bronchitis, asthma, and pneumonia (Hong et al., 2015; Pizzorno et al., 2016). The *H. helix* extract and isolated compounds exerted a spasmolytic effect in isolated guinea-pig ileum (Trute et al., 1997).

Clinical evidence. *Hedera helix* preparations have been trialed clinically for respiratory diseases such as bronchial asthma, and improvement of airway resistance, intrathoracic gas volume, and forced expiratory volume were evaluated (Hofmann et al., 2003; Guo et al., 2006; Holzinger and Chenot, 2011). However, despite its established traditional use, very few controlled clinical studies have been published. A randomized, double-blind trial was carried was conducted with an *H. helix* standardized extract. A total of 181 patients presenting acute cough was treated with 35 mg of the extract, three times daily, for 7 days. The group treated with ivy extract showed a clinically relevant reduction in cough score, the severity of symptoms associated with cough, and bronchitis, in comparison with the control group. The reduction of symptoms occurred in the first 48 h. of treatment. The observed adverse effects were non-serious, mild, or moderate severity and not related to the treatment (Schaefer et al., 2016). A study involving 139 patients having acute bronchitis and productive cough for at least three days, compared the treatment using a standardized extract of *H. helix* and acetylcysteine. No statistically differences were observed between the treatments. Moreover, both treatments showed to be safe and effective in children and adults (Kruttschnitt et al., 2019; Kruttschnitt et al., 2020). Moreover, a review of the treatment of upper respiratory tract infections identified 10 clinical trials, including three controlled trials (one of which was placebo-controlled) and 7 non-randomized observational studies (Holzinger and Chenot, 2011). Overall, the clinical evidence is High for bronchitis and the common cold.

Safety. Although gastrointestinal reactions (EMA, 2015a) and nausea, vomiting, and diarrhea have been listed among the symptoms of overdose (Bisset, 1994), and although there might be allergic reactions to the saponins, *H. helix* preparations are generally considered to be safe. There was a minimal report of adverse effects in 10 clinical trials, including three controlled trials (one of which was placebo-controlled) and 7 non-randomized observational studies (Holzinger and Chenot, 2011). Overall, the safety is High, and there are no reports to infer that the preparations from the plant may interfere negatively with the disease or NSAIDs.

Specific warnings and precautions of use. *Hedera helix* preparations are contraindicated if the patient presents dyspnea, fever, or purulent sputum. They should be used with care in patients suffering from gastritis or gastric ulcers (EMA, 2015a).

Overall assessment. *Hedera helix* preparations could be useful in the symptomatic relief of respiratory diseases by exerting an expectorant and anti-inflammatory effect on the respiratory tract. In many countries, licensed preparations are regulated for use in children. There is a robust body of clinical evidence that can be considered as High. This herbal medicine safety can be rated as High, due to the side effects that had been related only in an overdose situation.

#### *Justicia pectoralis* Jacq. [syn. *Dianthera pectoralis* (Jacq.) J.F.Gmel.] – Acanthaceae (Leaves)

Indications in the context of respiratory conditions. *Justicia pectoralis* (chambá) is indicated for symptoms of respiratory disease, as expectorant (BRASIL, 2011) and immunostimulant (CUBA, 2014).

Chemical composition. Coumarins (mainly coumarin and umbelliferone); flavonoids (e.g., quercetin, kaempferol, swertisin, and derivatives) (Lino et al., 1997; Oliveira et al., 2000); lignans (e.g., justicidin B) (Joseph et al., 1988).

Posology (based on traditional uses). 5 g in 150 ml of water as an infusion (BRASIL, 2011).

Preclinical evidence. This herbal medicine has been experimentally proven for symptoms of respiratory disease, namely asthma. A standardized hydroalcoholic extract of *J. pectoralis* presented inhibitory effects on the tracheal smooth muscle of rats subjected to challenge with ovalbumin (OVA) in an allergen model that reproduces many features of clinical asthma such as bronchial hyper-reactivity. Administered by gavage to sensitized rats after challenge with saline or OVA, the extract of *J. pectoralis* decreased the exacerbated responsiveness of rat trachea caused by the challenge with the sensitizing antigen. The oral administration of the standardized extract reduced the hyperresponsiveness in OVA-challenged trachea in preparations stimulated with KCl (potassium chloride) or ACh (acetylcholine). These effects on rat airways are possibly related to its anti-inflammatory activity, as observed by its ability to significantly inhibit the increase of the levels of TNF-α and IL-1β, pro-inflammatory cytokines, in bronchoalveolar lavage of OVA-challenged rats. These work findings reinforce the notion that the extract of *J. pectoralis* possesses potential anti-asthmatic properties (Moura et al., 2017). In another study, the aqueous extract of *J. pectoralis* was evaluated in sensitized guinea-pig. The crude extract reduced the formation of histamine-induced wheals. Moreover, the extract was tested on guinea-pig tracheal contraction caused by the cumulative dosing of histamine. *Justicia pectoralis* reduced histamine-induced tracheal smooth muscle contractions (Cameron et al., 2015).

Clinical evidence. *Justicia pectoralis* preparations have been trialed clinically for cough in children only. The efficacy of chambá syrup at 5% was evaluated in a randomized, double-blind clinical trial, in 114 children presenting cough and other respiratory symptoms. Chambá syrup was effective in relieving symptoms of cough, nasal congestion, and rhinorrhea, as well as improving the sleeping capacity of children when compared to placebo (Nascimento, 2018). Overall, the clinical evidence is High for cough.

Safety. In traditional doses, there is no report about the toxicity of *J. pectoralis*, including children.

Specific warnings and precautions of use. Due to the presence of coumarin and derivatives, *J. pectoralis* use should be avoided together non-steroidal anti-inflammatory or anti-coagulant drugs (Wittkowsky, 2003). Overall, safety is High.

Overall assessment. *Justicia pectoralis* preparations might be useful in the symptomatic relief of respiratory disease through exerting an anti-inflammatory effect on the respiratory tract. The clinical evidence may be considered High. Even though chambá is known to have potential antiplatelet effects, overall, it can be considered presenting Medium safety.

#### *Magnolia officinalis* Rehder & E.H. Wilson - Magnoliaceae (Bark)

Indications in the context of respiratory conditions. *Magnolia officinalis* is indicated for symptoms of respiratory disease, namely cough, fever, and shortness of breath (WHO, 2009).

Chemical composition. Biphenyl neolignan derivatives: magnolol (5,5’-diallyl-2,2’-dihydroxy biphenyl) and honokiol (5,3’-diallyl-2,4’-dihydroxy biphenyl); isoquinoline-type alkaloids, the majority of which are aporphine (*N*-methylcoxylonine, (*S*)-magnoflorine, magnofficine, (*R*)-asimilobine, corytuberine, anonaine, liriodenine) and benzylisoquinoline derivatives (magnocurarine, (*S*)-tembetarine, lotusine, (*R*)-oblongine, reticuline); essential oil (the major constituents are α-, β-, and γ-eudesmol) (Yan et al., 2013; Poivre and Duez, 2017).

Posology (based on traditional uses). 3-9 g of crude drug (decoction) daily in divided doses (WHO, 2009).

Preclinical evidence. This herbal medicine has been experimentally proven for asthma. Studies in animals and *in vitro* models have demonstrated multiple biological properties of honokiol, including anti-asthma (through IL4 and IFN-NF-kB) (Hong et al., 2018). Other related symptom studies are anti-histamine (Shin et al., 2001) and anti-HIV (human immunodeficiency viruses) activities (Amblard et al., 2006).

Clinical evidence. Magnolia preparations have been trialed clinically for asthma. Preliminary clinical studies showed the benefits of magnolia for oral health (Campus et al., 2011). In non-comparative research, as add-on therapy in 148 patients with mild to moderate asthma using inhaled corticosteroids, an extract of Magnoliae flos had a beneficial effect on asthma control (Park et al., 2012). Overall, the clinical evidence is Medium for asthma.

Safety. Magnolol and honokiol are the main ingredients of magnolia bark and, therefore, were considered directly relevant to evaluate the safety of the product. Full clinical monitoring, including biochemical and hematological analysis, showed no evidence of toxicity reported in subjects consuming magnolia bark containing supplements at a dose of 750 mg/person/day (approximately 15 and 60 mg/person per day of honokiol and magnolol respectively) for 42 days (Garrison and Chambliss, 2006). According to other studies, safety and toxicity of magnolia bark do not differ notably but may reflect insufficiencies of these toxicity studies. Therefore, currently, studies of the mechanisms of toxicity of magnolia bark are insufficient (Luo et al., 2019). However, in the context of COVID-19, magnolol has been identified as a potential enhancer of ACE2 expression (ANSES, 2020). Overall, the safety of *M. oficinalis* is Medium.

Specific warnings and precautions of use. None known.

Overall assessment. Magnolia bark has been used in Chinese and Japanese traditional medicines for the treatment of asthma, allergic disease, as well as for the alleviation of headaches, muscular pain, and fever (Forrest, 1995; Lee et al., 2011). As *M. officinalis* preparations are not clinically proven to provide symptomatic relief of flu symptoms and its active principle (magnolol) may enhance the entry SARS-CoV-2 virus into the cell, the clinical evidence may be considered Medium and the safety, in the COVID-19 context, Medium.

#### *Malva sylvestris* L. – Malvaceae (Leaves)

Indication in the context of respiratory conditions. *Malva sylvestris* is indicated for respiratory disease, namely oral or pharyngeal irritations and dry cough (BRASIL, 2011; EMA, 2018d).

Chemical composition. Mucilages (mainly glucuronic and galacturonic acids, rhamnose, galactose, fructose, glucose, trehalose); flavonoids (e.g., malvidin, delphinidin, myricetin, apigenin, kaempferol, genistein, and derivatives); tannins (Farina et al., 1995; Paul, 2016); hydroxycinnamic acid and derivatives; benzoic acid and derivatives; monoterpenes (Cutillo et al., 2006).

Posology (based on traditional uses). 1.8 g in 150 ml as infusion or decoction three times daily (EMA, 2018d).

Preclinical evidence. *Malva sylvestris* preparations have been experimentally studied for cough. The anti-tussive activity of its mucilage and isolated rhamnogalacturonan was evaluated in cats. Both substances suppressed the cough reflex and decreased the frequency of cough, especially in the laryngopharynx area, although the polysaccharide was more active than mucilage (Nosalova et al., 2005). Another study showed anti-inflammatory and analgesic action through traditional pharmacological *in vivo* models (Seddighfar et al., 2020). Mucilage seems to be responsible for the cough suppressive activity of this species (Čapek et al., 1999). Another experimental related activity is anti-inflammatory. *Malva sylvestris* ethanolic extract presented anti-inflammatory activity in mice through the ear edema model. The extract reduced the levels of IL-1β in tissue challenged by TPA. Also, the extract and isolated compounds inhibited myeloperoxidase activity. Malvidin-3-glucoside presented inhibitory activity similar to dexamethasone (Prudente et al., 2013). The aqueous extract can suppress the expression of several pro-cytokine genes such as TNF-α, IL-1β, COX-2, iNOS (Mirghiasi et al., 2015).

Clinical evidence. *Malva sylvestris* has not been trialed clinically for respiratory disease. Overall, the clinical evidence is Low.

Safety. In traditional doses, there is no report about the toxicity of *M. sylvestris*. However, high doses may cause nausea, vomiting and diarrhea, nervous excitement, and insomnia (BRASIL, 2015a; BRASIL, 2019b). Overall, safety is High.

Specific Warnings and precautions of use. In general, mucilage can alter the absorption of some drugs (BRASIL, 2015a).

Overall assessment. *Malva sylvestris* has been traditionally used as cough therapy and may be useful in the relief of COVID-19 symptoms through exerting a soothing effect on the respiratory tract. The clinical evidence may be considered Low, but this herbal medicine is considered presenting High safety.

#### *Mikania glomerata* Spreng. and *M. laevigata* Sch.Bip. ex Baker Asteraceae (Leaves)

Indications in the context of respiratory conditions. *Mikania glomerata* is indicated for symptoms of respiratory disease, namely cough, and as expectorant (BRASIL, 2011; BRASIL, 2017; BRASIL, 2018).

Chemical composition. Essential oil (germacrene D and β-caryophyllene as the major components) (Ueno and Sawaya, 2019); coumarins (e.g., coumarin, trans-*o*-coumaric acid),; phenolic acids (e.g., chlorogenic and caffeoylquinic acids) (Lazzari Almeida et al., 2017); terpenoids and steroids (e.g., friedelin, ent-kaurenoic acid, ent-kaur-16(17)-en-19-oic acid, ent-beyer-15(16)-en-19-oic acid, ent-15β-benzoyloxykaur16(17)-en-19-oic acid, grandifloric acid, ent-cinnamoylgrandifloric acid, ent-benzoylgrandifloric acid 17-hydroxy-ent-kaur-15(16)-en-19-oic acid, stigmasterol, β-sitosterol) (Veneziani and Oliveira, 1999; Bertolucci et al., 2013).

Posology (based on traditional uses). 3 g of dried leaves in 150 ml, as an infusion, 2 times daily. However, the use of dosage forms, with defined composition, is recommended.

Preclinical evidence. This herbal medicine has been experimentally proven for cough. The aqueous extract reduced the contractile effect of histamine on the isolated guinea-pig trachea and human bronchi (Moura et al., 2002). A fraction of *M. glomerata* ethanolic extract was evaluated through an allergic pleurisy model in rats and resulted in the inhibition of leukocyte infiltration (Fierro et al., 1999).

Clinical evidence. *Mikania glomerata* preparations have been trialed clinically for one of the related diseases, namely cough. This herbal medicine has been used in Brazil in respiratory diseases, such as asthma, cough, and throat inflammation (Agra et al., 2008; Brandao et al., 2009). A clinical trial sponsored by the Brazilian government showed that the infusion of *M. glomerata* (5, 10, and 15 g in 200 ml of water) had an unequivocal bronchodilator action and evident dose-dependent antitussive effect (Amaral et al., 2006). A randomized study involving 62 patients compared the bronchodilator effect of *M. glomerata* syrup and salbutamol inhaler and found *M. glomerata* syrup did not present effect (Garcia et al., 2020). However, the overall problem with this study refers to the differences between the investigated pharmaceutical preparations. Overall, the clinical evidence is High for asthma, but Low for other types of cough.

Safety. Some non-clinical studies report its safety. *Mikania glomerata* hydroalcoholic (70%) extract presented LD_50_ ~ 3000 mg/kg and did not produce any biochemical, hematological, and morphological changes in mice (Santana et al., 2013). Moreover, *M. glomerata* did not present genotoxicity, mutagenicity, teratogenicity or antifertility activity (Sa et al., 2003; Sá et al., 2006; Barbosa et al., 2012; Fulanetti et al., 2016). Doses above those recommended may cause vomiting and diarrhea (Matos, 2000; BRASIL, 2011). *Mikania glomerata* syrup is included in the Brazilian List of Essential Medicines, and so far, there are no reported concerns relating to safety from the point of view of pharmacovigilance.

Specific Warnings and precautions of use. Not to be used while under treatment with NSAIDs. The use may interfere with coagulation due to the presence of coumarin derivatives (Matos, 2000; Wittkowsky, 2003; BRASIL, 2011). Overall, safety is Medium.

Overall assessment. *Mikania glomerata* is in the list of essential medicine in Brazil for the symptomatic relief of cough and asthma and has been prescribed for children for several years without a pharmacovigilance report. Preclinical and clinical data back up this indication, although guaco is not clinically proven to provide relief of flu symptoms. The clinical evidence may be considered High. Even though guaco is known to have potential antiplatelet effects, overall, it can be considered presenting Medium safety.

#### *Ocimum gratissimum* L. -Lamiaceae (Leaves)

Indication in the context of respiratory conditions. *Ocimum gratisssimum* is indicated for symptoms of cold, influenza, fever, asthma, bronchitis (WHO, 2002).

Chemical composition. Essential oil (eugenol, thymol, and 1,8-cineol as the major components) (Prabhu et al., 2009; Monga et al., 2017); phenolics (quercetin and derivatives, luteolin and derivatives, kaempferol and derivatives, catechin, epi-catechin, cafeic acid); triterpenes (ursolic acid) (Prabhu et al., 2009; BRASIL, 2015b; Siva et al., 2016; Monga et al., 2017).

Posology (based on traditional uses). 1-3 g in 150 ml of boiling water, as an infusion, 3-4 times daily (BRASIL, 2019b).

Preclinical evidence. This herbal medicine has been experimentally proven for cough. An aqueous extract of *O. gratissimum* was evaluated in OVA-sensitized guinea-pig about anti-tussive and anti-asthma activities. The aqueous extract did not exert effect in pre-convulsive dyspnea; however, reduced in a significant manner, the volume of tracheal liquid, although it did not interfere in the fluid viscosity, and reduced in more than 80% the number of cough episodes (Ozolua et al., 2016). Another experimental related activity is anti-inflammatory. The essential oil of *O. gratissimum* presented an antinociceptive effect in mice, in evaluation using classical methods (Rabelo et al., 2003; Paula‐Freire et al., 2013). A flavonoid-rich fraction of *O. gratissimum*, in mice, presented anti-inflammatory activity, reducing the number of the leucocytes in the peritoneum, and inhibited iNOS, COX-2 (in the same degree than indomethacin). Also, inhibited LPS-induced NO, IL-1β, and TNF-a production in RAW 264.7 cells (Ajayi et al., 2017).

Clinical evidence. *Ocimum gratissimum* has not been trialed clinically for respiratory diseases. Overall, the clinical evidence is Low.

Safety. In traditional doses, there is no report about the toxicity of *O. gratissimum*.

Specific Warnings and precautions of use. Due to the presence of estragole, a naturally occurring genotoxic carcinogen, *O. gratissimum* should be used for a short time, in the recommended posology (EMA, 2005) (see also *Foeniculum vulgare*).

Overall assessment. Its traditional use as cough therapy in the context of upper respiratory conditions is not backed up by clinical data profile, but its preclinical evidence and safety profile may allow the potential use in the relief of early symptoms of COVID-19. The clinical evidence is Low, and although there are some concerns about the presence of estragole, in the traditional doses, this herbal medicine is considered presenting High safety.

#### *Pelargonium sidoides* DC and/or *Pelargonium reniforme* Curt. -Geraniaceae (Root)

Indications in the context of respiratory conditions. *Pelargonium sidoides* is indicated for the common cold (EMA, 2018c), cough, and bronchitis (COLOMBIA, 2008). Umkaloabo preparations are available in some European Countries with a full marketing authorization (e.g., Bulgaria, Czech Republic, Germany, Latvia) or register as a traditional herbal medicinal product (e.g., Austria, Hungary, Italy, The Netherlands, Poland, Spain, Sweden), and are widely used for ‘acute bronchitis’ and other respiratory infections’ (EMA, 2018c). Practically all research has been conducted with the proprietary extract EPs 7630^®^. The species originate from Southern Africa, and the term Umkaloabo is claimed to be a fusion of two terms from Zulu (“Umkuhlune” - coughing and fever; and “Uhlabo” = pain in the chest) (Heinrich et al., 2018).

Chemical composition. Relevant key metabolites assumed to be active are hydrolyzable tannins, (+)-catechin, gallic acid, and methyl gallate, including some unusual *O*-galloyl-C-glucosyl flavones., scopoletin, 6,8-dihydroxy-5,7-dimethoxycoumarin, 5,6,7-trimethoxycoumarin. Other coumarins, as well as, quercetin 3-*O*-β-D-glucoside, myricetin, and other flavonoids have been isolated from both species. *Pelargonium sidoides* yielded some benzopyranones. Umckalin is only known from *P. sidoides* (Maree and Viljoen, 2012); pelargoniins (an ellagitannin) and the diterpene reniformin have been identified in *P. reniforme* (Edwards et al., 2015).

Posology. Oral drops, tablets, and syrups are most commonly used (EMA, 2018c). Commercial preparations with regulated quality, a defined composition, and an adequate dose are available on some markets globally.

Preclinical evidence. This herbal medicine has been experimentally proven for anti-viral activity. The commercial extract EPs 7630^®^ interfered *in vitro* with the replication of different respiratory viruses, including human coronavirus (Michaelis et al., 2011), *Influenza* virus (*in vitro* and *in vivo*) (Theisen and Muller, 2012), and *Rhinovirus* isolated from patients with severe asthma (Roth et al., 2019). EPs 7630 was able to stimulate IFN-β *in vitro*, and gallic acid enhanced the expression of iNOS and TNF-α (Kolodziej et al., 2003).

Clinical evidence. *Pelargonium sidoides* preparations have been trialed clinically for cough. In a randomized, controlled trial, involving 124 adults presenting acute bronchitis, were treated with 30 drops of EPs 7630^®^, for seven days. The treated group showed a significant improvement regarding the Bronchitis Severity Score (BSS) compared with the placebo group (Chuchalin et al., 2005). In a Cochrane review, the efficacy of *P. sidoides* preparations in acute respiratory infections in RCT was assessed and indicated that it might be useful in the alleviation of symptoms of acute rhinosinusitis and the common cold in adults. However, the authors also raised concerns regarding the studies’ quality (Timmer et al., 2013). Similarly, Schapowals’ meta-analyses of randomized, double-blind, placebo-controlled trials concluded that it is useful in the management of the common cold (Schapowal et al., 2019). Overall, the clinical evidence is High for bronchitis and the common cold.

Safety. Umkaloabo preparations are generally considered to be safe, although gastrointestinal discomfort (stomach pain, heartburn, nausea, or diarrhea) might occur with no major concerns about interaction risks (Edwards et al., 2015). However, due to the potential immunomodulatory activity, overall, safety is Medium.

Specific warnings and precautions of use. As with all other products discussed here, there is no evidence for specific therapeutic benefits, and it is important to communicate this to potential users. The safety concerns listed above need to be kept in mind.

Overall assessment. Umkaloabo may be useful in the symptomatic relief of respiratory symptoms through exerting a soothing effect on the respiratory tract. A relatively large number of clinical studies and a series of meta-analyses provide evidence that Umkaloabo preparations seem to be efficacious both in the treatment (reducing symptoms and duration) and prevention of the common cold. However, it must be discontinued to avoid immunostimulation in complications in later phases of the disease. The clinical evidence is High, and this herbal medicine is considered presenting Medium safety.

#### *Pimpinella anisum* L. – Apiaceae (Fruits)

Indications in the context of respiratory conditions. *Pimpinella anisum* is indicated for cough and fever (WHO, 2009; CHILE, 2010) and as antispasmodic (WHO, 2009; CHILE, 2010; BRASIL, 2011).

Chemical composition. Essential oil (*trans*-anethole as the main compound) (Orav et al., 2008); chlorogenic acid derivatives; flavonoids (orientin, vitexin, and others, coumarin derivatives; triterpenes and steroidal compounds (Zobel et al., 1991; Reichling and Galati, 2004; Abdollahi Fard, 2012).

Posology (based on traditional uses). 1,5 g of the dried fruits in 150 ml, as an infusion, three times daily (BRASIL, 2011; Barnes et al., 2012; EMA, 2013b).

Preclinical evidence. This herbal medicine has been experimentally proven for cough. Aqueous and ethanol extracts of *P. anisum* presented myorelaxant effect in isolated tracheal chains of guinea-pig. The relaxant effect was similar to promoted by theophylline. Interesting to note that the essential oil did not present a significant effect (Boskabady and Ramazani-Assari, 2001).

Clinical evidence. *Pimpinella anisum* preparations have been trialed clinically for asthma. The bronchodilator activity of *P. anisum* was evaluated in a study involving 50 patients presenting bronchial asthma. The patients ingested tea (2 g in 200 ml of water), twice a day for 40 days. All patients presented a reduction of cough episodes after 21 days of treatment (from more than 6 episodes/day to none), as well as dyspnea and wheezing. Also, an improvement in the breath-holding time and respiratory rate was observed (Paheerathan, 2019). Overall, the clinical evidence is High for asthma-related cough and Low for cough and fever.

Safety. In the traditional doses, there is no report about the toxicity of *P. anisum*. However, allergic reactions can occur, for example, rhinoconjunctivitis (García-González et al., 2002; EMA, 2013b). Overall, safety is High.

Specific warnings and precautions of use. The concomitant use with anti-coagulant drugs should be avoided due to the presence of coumarins (Dinehart and Henry, 2005).

Overall assessment. Its traditional use as cough therapy in the context of upper respiratory conditions is not backed up by robust clinical data profile, but its safety profile allows for potential use in the relief of early symptoms of COVID-19. The clinical evidence may be considered High. Even though *P. anisum* has potential antiplatelet effects, overall, it can be considered presenting Medium safety.

#### *Plantago lanceolata* L. – Plantaginaceae (Leaves)

Indication in the context of respiratory conditions. *Plantago lanceolata* is indicated for symptoms of respiratory disease, namely cough, pharyngitis, and fever (CHILE, 2010). Other indications include as demulcent, to reduce bronchial catarrh and mild inflammation of the pharynx (Blumenthal et al., 1998; ESCOP, 2003b; Wichtl, 2004; Boskabady et al., 2006a).

Chemical composition. Carbohydrates (Jiang et al., 2019); flavonoids (apigenin, luteolin and their derivatives) (Haddadian and Zahmatkash, 2014; Ferrazzano et al., 2015); iridoid glycosides (aucubin and catalpol as the main ones) (EMA, 2014b; Lotter et al., 2017; Hasan et al., 2018); phenylethanoids (Bahadori et al., 2020); saponins (Wichtl, 2004); tannins (Wichtl, 2004; Grigore et al., 2015); and essential oil (amyl vinyl carbinol (E),4(3-oxo-2,6,6-trimethylcyclo-hex-2-en-1-yl)-3-buten-2-ol, 6-(3-hydroxy-1-butenyl)-1,5,5-trimethyl-7-oxabicyclo[4,1,0]heptan-3-ol, and benzoic acid as major components) (Fons et al., 1998; Bajer et al., 2016).

Posology (based on traditional uses). 2 g of leaves in 150 ml boiling water, two to three times daily; 160-190 mg of powdered plant coated tablet or lozenges, maximum dose of 1.28 g daily (EMA, 2014b). It is preferable to use a commercial preparation with a defined composition and an adequate dose (EMA, 2014b).

Preclinical evidence. This herbal medicine has been experimentally proven for cough. An ethanol extract of *P. lanceolata* reduces the number of coughs provoked by citric acid, in guinea-pig, in a similar way to codeine (Boskabady et al., 2006a). Other experiments showed that an ethanol extract of this species inhibited the barium-induced contraction of guinea-pig isolated trachea (Fleer and Verspohl, 2007). The pharmacological effects described in literature support both the oral and oromucosal traditional use of herbal preparations of *P. lanceolata* as a demulcent for the symptomatic treatment of oral and pharyngeal mucosa irritation, associated to dry cough (Ortiz de Urbina et al., 1994; Herold et al., 2003; Vigo et al., 2005; Hausmann et al., 2007).

Clinical evidence. *Plantago lanceolata* has not been trialed clinically for respiratory tract disorders. However, there is some evidence in the literature for the traditional internal use of *P. lanceolata* as a mucilage in the treatment of irritations of oral and pharyngeal mucosa and associated dry cough (Wegener and Kraft, 1999). However, data on this clinical assay are scarce. Overall, the clinical evidence is Low.

Safety. Currently, there is a lack of toxicity and clinical safety data for *P. lanceolata*, which warrants further investigation for this plant. However, popular use points to hypersensitivity reactions as the main side effects reported (Ozkol et al., 2012; EMA, 2014b). Overall, the safety is High as there are no reports to infer that the preparations may interfere with the disease or NSAIDs.

Specific warnings and precautions of use. The topical use of this plant is not recommended (EMA, 2011a; Ozkol et al., 2012; EMA, 2014b). *Plantago lanceolata* pollen may cause hayfever, especially if the plant is abundant in a region like in many subtropical regions (Calabozo et al., 2001). Pla 11 has been named the main allergen with IgE-binding capacity present in this plant, although other IgE-binding proteins have also been identified (Sousa et al., 2014). Treatment should not exceed one week (EMA, 2014b). Minor diarrhea has also been reported (EMA, 2011a).

Overall Assessment. Although *P. lanceolata* is not clinically proven to provide symptomatic relief of flu symptoms, there are enough preclinical evidences to allow the use of this herbal medicine to relieve respiratory symptoms through exerting an expectorant and anti-inflammatory effect. The clinical evidence is Low, but this herbal medicine is considered presenting High safety.

#### *Platycodon chinensis* (Jacq). A.DC. [syn. *Platycodon grandiflorus* (Jacq.) A.DC.] – Campanulaceae (Roots)

Indications in the context of respiratory conditions. *Platycodon chinensis* is indicated for cough (WHO, 1999). Other related indications include expectorant (WHO, 1999); upper respiratory infections; sore throat (PRC, 1992; Li Y. H. et al., 2013).

Chemical composition. Triterpene saponins (2%) (e.g., platycodigenin; polygalacic acid) ((Fu et al., 2011); steroids (δ-stigmasterol, α-spinasterin, betulin) (Wagner H. et al., 2015); essential oil (He et al., 2013).

Posology (based on traditional uses). 2–9 g in 150 ml as a decoction, daily (Chang and But, 1987).

Preclinical evidence. *Platycodon chinensis* has been experimentally proven for cough and fever in mice (Oh et al., 2010; Zhang et al., 2015). An aqueous extract from *P. chinensis* presented anti-inflammatory activity in rats, in the carrageenan model. The extract inhibited the production of cytokines and the expression of COX-2 (Kim et al., 2006). Platycosides are inhibitors of the production of IL-6, PGD2, LTC4, and β-Hex, therefore presenting potential for allergy symptoms treatment (Oh et al., 2010). Crude platycodin (80 mg/kg, p.o.) presented expectorant activity, higher than ammonium chloride, in guinea-pig. The author showed that crude platycodin also presented analgesic and antipyretic activities in mice (Lee, 1975). Crude platycodin presented anti-histaminic and anticholinergic effects in guinea-pig, but not presented anti-serotonin or anti-bradykinin effect (Takagi and Lee, 1972). Isolated platycodin D and D3 presented anti-inflammatory and potential expectorant activities. In rat and hamster tracheal surface epithelial cells, both compounds increased mucin release. Platycodin D3 was most active than ATP (mucin secretagogue) and ambroxol (mucolytic) (Shin et al., 2002). A decoction of *P. chinensis* inhibited cough in mice (Zhang et al., 2015).

Clinical evidence. This herbal medicine has been trialed clinically for pneumonia. Two patients presenting pneumoniae were treated with aqueous extract of *P. chinensis* and acupuncture. Patients presented improvement in cough, phlegm, and fever. Chest X-ray showed a decrease in lung infiltration (Kim et al., 2001). Overall, the clinical evidence is Low.

Safety. *Platycodon chinensis* was considered safe in preclinical evaluation. Platycodins present hemolytic activity; however, as they are poorly absorbed, the oral ingestion in the suggested doses seems to be safe (WHO, 1999). In the context of COVID-19, overall, the safety is High, as there are no reports to infer that such herbal preparations may interfere with the disease or NSAIDs.

Specific warnings and precautions of use. Radix *Platycodi* reportedly depresses the central nervous system (CNS) (Lee, 1975; WHO, 1989). Therefore, the concomitant use of this herbal medicine with alcohol or hypnotic and sedative drugs should be avoided. Overall, the safety is Medium, but there are no reports to infer that such herbal preparations may interfere with the disease or NSAIDs.

Overall assessment. Based on traditional use, preparations could be useful in respiratory symptoms relief, through exerting an antitussive effect, although it is not backed up by robust clinical data. The clinical evidence is Low, but this herbal medicine is considered presenting High safety.

#### *Polygala senega* L. – Polygalaceae (Roots)

Indication in the context of respiratory conditions. *Polygala senega* is indicated for cough (WHO, 2002; ESCOP, 2003b). Other related indications include its actions as expectorant bronchitis, emphysema, and catarrh of the upper respiratory tract (Briggs, 1988; WHO, 2002; ESCOP, 2003b; Lacaille-Dubois et al., 2020).

Chemical composition. Organic acids (salicylic acid and its methyl ester) (Bradley, 1992; COE, 2008); monosaccharides (Takiura et al., 1974; Takiura et al., 1975); oligosaccharides (Ikeya et al., 1991; Saitoh et al., 1994); acylated glucosides (Ikeya et al., 1991; Ikeya et al., 1994); triterpene saponins (Tsukitani et al., 1973; Tsukitani and Shoji, 1973; Sakuma and Shoji, 1981; Sakuma and Shoji, 1982; Bradley, 1992; Yoshikawa et al., 1995b; Yoshikawa et al., 1995a; Yoshikawa et al., 1996; Wichtl, 2004; COE, 2008; Evans, 2009); essential oils (Sakuma and Shoji, 1982; COE, 2008), flavonoids; coumarins (Lacaille-Dubois et al., 2020); and xanthones (Ikeya et al., 1994).

Posology (based on traditional uses). Herbal tea (dried root): 0.5–1.0 g three times a day (BHMA, 1983; Bradley, 1992). It is preferable to use a commercial preparation with a defined composition and an adequate dose.

Preclinical evidence. This herbal medicine has not been experimentally proven for symptoms of respiratory disease. However, the expectorant activity of the crude drug is due to the constituent saponins, which produce local irritation of the mucous membranes of the throat and respiratory tract. This irritation stimulates an increase in bronchial secretions, thereby diluting the mucus, reducing its viscosity and facilitating expectoration (Boyd and Palmer, 1946; Misawa and Yanaura, 1980; Reynolds and Parfilt, 1996). Isolated saponins from roots of this species were able to enhance anti-OVA specific IgG and IgG2a in mice (Katselis et al., 2007).

Clinical evidence. *Polygala senega* preparations have not been trialed clinically for respiratory diseases. French patent mentioned that a triterpene acid isolated from *P. senega* possesses anti-inflammatory action (Mitchell and Rook, 1979). Another French Patent (Tubery P., Fr Demande Patent 2,202,683) claimed the fluid extract prepared with *P. senega* could reduce the viscosity of phlegm in patients with bronchiectasis (Barnes et al., 2012). However, data on this clinical trial are limited. Overall, the clinical evidence is Low.

Safety. Generally, there is a lack of toxicity data and clinical safety for *Polygala* species. Saponins of this species are usually irritant to the gastrointestinal mucosa (Mitchell and Rook, 1979), and large doses of the plant are reported to cause nausea, diarrhea, and vomiting (Foster and Tyler, 1999; WHO, 2002). In the context of COVID-19, overall safety is High, as there are no reports to infer that the preparations may interfere with the disease or NSAIDs.

Specific warnings and precautions of use. Due to the lack of toxicity data and potential risks associated with chronic ingestion of this herbal medicine, the use by patients with gastric ulcer or gastric inflammation should be avoided. Chronic exposure of the intestinal mucosa to saponins may also inhibit active nutrient absorption (Capasso et al., 2003).

Overall assessment. Senega preparations might be useful in the symptomatic relief of respiratory symptoms through exerting an antitussive effect. Although the clinical evidence is Low, this herbal medicine is considered presenting High safety.

#### *Polypodium vulgare* L. – Polypodiaceae (Rhizomes)

Indication in the context of respiratory conditions. *Polypodium vulgare* is indicated for symptoms of respiratory disease, namely cough (EMA, 2008c).

Chemical composition. Flavonoids (Grzybek, 1976; Karl et al., 1982); hydroxycinnamic acids (caffeic acid 4’-glucoside, 0.6% in the rhizome) (Jizba and Herout, 1967; Grzybek, 1976); phytoecdysteroids (Arai et al., 1991; Yamada et al., 1992; Marco et al., 1993; Coll et al., 1994; Reixach et al., 1996; Messeguer et al., 1998; Simon et al., 2011); triterpenes (Devys et al., 1969; Robinson et al., 1973; EMA, 2008b).

Posology (based on traditional uses). 4–5 g, as a decoction, 3–4 times a day. Not to be taken for more than a week (EMA, 2008c).

Preclinical evidence. This herbal medicine has been experimentally proven for respiratory disease. A hydroalcoholic extract presented a myorelaxant dose-dependent effect in rabbit isolated tracheal preparation. The effect was cholinergic-like (Naz et al., 2016). However, the expectorant and antitussive effects of saponins are known (Hostettmann and Marston, 2005), and the saponin in the fresh plant of polypody acts as a cough suppressant (Høeg, 1984).

Clinical evidence. *Polypodium vulgare* has not been trialed clinically for respiratory diseases. Overall, the clinical evidence is Low.

Safety. Overall, the safety is High as there are no reports to infer that the preparation from this plant may interfere with the disease or NSAIDs.

Specific warnings and precautions of use. A mild laxative effect may happen when this plant is used as expectorant (EMA, 2008c). Moreover, due to possible hypotensive and hypnotic effects, patients should be aware of potential interactions (Ulbricht et al., 2006).

Overall assessment. *Polypodium vulgare* preparations could be useful in the relief of the respiratory symptoms, through exerting an antitussive effect. Although the clinical evidence is Low, this herbal medicine is considered presenting High safety.

#### *Potentilla erecta* (L.) Raeusch. – Rosaceae (Rhizomes, Roots)

Indications in the context of respiratory conditions. *Potentilla erecta* is indicated for cough and symptomatic treatment of minor inflammations of the oral mucosa (EMA, 2010; ESCOP, 2013).

Chemical composition. Hydrolysable tannins (pedunculagin, pentadigalloylglucose, agrimoniin, laevigatin B, tormentillin); condensed tannins/proanthocyanidins ([4,6]-all-trans-bi-(+)-catechin = procyanidin B6, [4,8]-2,3-trans-3,4-cis-bi-(+)-catechin, [4,8]-all-trans-bi-(+)-catechin = procyanidin B3), [6′,6]-all-trans-bi-(+)-catechin, procyanidin B1, procyanidin B2, procyanidin B5, (+)-catechin-[4,8]-(+)-catechin-[4,8]-(+)-catechin, (+)-catechin-[6′,8]-(+)-catechin-[4,8]-(+)-catechin; triterpenoids: 2α,3β-dihydroxyurs-12 -en-28-oic acid β-D-glucopyranosyl ester, 3-epi-pomolic acid 28-*O*-β-D-glucoside, 3β,19α-dihydroxyolean-12-en-28-oic acid β-D-glucopyranosyl ester, 3β,19α-dihydroxyurs-12-en-28-oic acid β-D-glucopyranosyl ester, arjunetin, chinovic acid, kaji-ichogoside F1, tormentic acid, tormentoside (Tomczyk and Latté, 2009; Melzig and Böttger, 2020).

Posology (based on traditional uses). 1.5–2.0 g per 100 ml of water, as an infusion, or 0.8–3.0 g per 100 ml of water, as a decoction. The preparation should be used to rinse the mouth several times daily (EMA, 2010). It is preferable to use a commercial preparation (e.g., tincture) with a defined composition and an adequate dose.

Preclinical evidence. This herbal medicine has not been experimentally proven for symptoms of respiratory disease. However, a moderate antiviral effect against *Herpes viridae* has been demonstrated for extracts of *P. erecta* and hydrolyzable and condensed tannins isolated from this plant. In particular, the antiviral activity against *Herpes virus* types I and II was reported, as well as the cytotoxic activity against *Influenza* virus type A2 and Cowpox (May, 1978). An agrimoniin-rich ethanolic extract of *P. erecta* inhibited the NF-kB and formation of IL-6, and PGE2 in TNF−α stimulated HaCaT cells (Wölfle et al., 2017). An animal test system with mice supported the demonstrated antiviral effects against the *Vaccine* virus together with an induction of interferon synthesis (May, 1978; Lund and Rimpler, 1985).

Clinical evidence. *Potentilla erecta* has not been trialed clinically for respiratory disease. Overall, the clinical evidence is Low.

Safety. Due to the limited studies in humans, animal models are used as alternatives to evaluate the safety of *P. erecta*. Acute toxicity of an aqueous extract of tormentil was assessed in animals using a single oral dose of 2.5 and 6.8 g/kg. No apparent toxic effect was observed. *Potentilla erecta* extracts should be considered safe for acute toxicity when also applied to humans (Shushunov et al., 2009).

Special warnings and precautions of use. Due to thrombolytic activity (Melzig and Böttger, 2020), caution should be taken to avoid interaction with thrombolytic drugs. Therefore, safety is Medium.

Overall assessment. *Potentilla erecta* preparations reportedly have relevant anti-viral effects, but the main uses are for buccal sores and inflammation. It is not established that it has a specific effect on the respiratory tract. The clinical evidence is Low, but this herbal medicine is considered presenting Medium safety.

#### *Primula veris* L. - Primulaceae (Roots)

Indications in the context of respiratory conditions. *Primula veris* is indicated for some symptoms of respiratory diseases, namely cough (EMA, 2012b).

Chemical composition. Saponins (e.g., primulasaponin I, primulasaponin II) (Müller et al., 2006); flavonoids (e.g., quercetin, luteolin, kaempferol, and isorhamnetin derivatives); phenolic acids (e.g., chlorogenic acid) (Bączek et al., 2017).

Posology. 0.2–0.5 g in 150 ml of boiling water, as an infusion, 3 times daily (EMA, 2012b). It is preferable to use a commercial preparation with a defined composition and an adequate dose.

Preclinical evidence. This herbal medicine has not been experimentally proven for symptoms of respiratory diseases. However, a *P. veris* dry extract presented potential anti-inflammatory activity *in vitro*, reducing IFN-γ, and inhibited prostaglandin and leukotriene synthesis. Also, inhibited the replication of human rhinovirus (HRV) and respiratory syncytial virus (RSV) (Seifert et al., 2012). A hydroalcoholic extract of *P. veris* presented anti-inflammatory activity in rats in the carrageenan-induced paw edema test (Marchyshyn et al., 2017).

Clinical evidence. *Primula veris* has not been trialed clinically for respiratory diseases. Overall, the clinical evidence is Low.

Safety. Cases of nausea, vomiting, gastric disturbances, and allergies may occur in doses higher than recommended (EMA, 2012b; Ghédira and Goetz, 2017). Overall, safety is High.

Special warnings and precautions of use. Concerns were raised over the concomitant use of *P. veris* and warfarin (Řehulková, 2001).

Overall assessment. Although *Primula veris* is not clinically proven to provide symptomatic relief of flu symptoms, it could be useful in the relief of the respiratory symptoms through an anti-inflammatory effect. The clinical evidence is Low, but this herbal medicine is considered presenting Medium safety.

#### *Salix alba* L., *Salix* sp. – Salicaceae (Cortex)

Indications in the context of respiratory conditions. *Salix alba* is indicated as antipyretic (BRASIL, 2011; EMA, 2017d); Other indications are as anti-inflammatory, and in the treatment of the flu and common cold (WHO, 2009).

Chemical composition. It is generally standardized to salicin. However, other salicylates, polyphenols, and flavonoids may also play important roles in therapeutic actions (Shara and Stohs, 2015). Some components: (+)-catechin, syringin, triandrin, ampelopsin, ethyl-1-hydroxy-6-oxocyclohex-2-enecarboxylate taxifolin, 3-*O*-methyltaxifolin, 7-*O*-methyltaxifolin-3-*O*-glucoside; *p*-coumaric, benzoic, *p*-hydroxybenzoic, *p*-methoxy benzoic, cinnamic, trans-p-methoxycinnamic, and cis-p-methoxy cinnamic acids (Agnolet et al., 2012).

Posology (based on traditional uses). 3 g dry stem barks in 150 ml as a decoction, 2–3 times a day (BRASIL, 2011). 1–3 g of the stem bark in 150 ml of boiling water three times daily (EMA, 2017d).

Preclinical evidence. This herbal medicine has been experimentally proven for fever. Its efficacy and safety have been reported in several studies. *Salix alba* extract showed significant analgesic as well as anti-inflammatory properties in mice and showed more potency than the standard drug aspirin in all the doses tested (Gyawali et al., 2013).

Clinical evidence. *Salix alba* preparations have been trialed clinically for fever and inflammation. A randomized study with 210 patients was conducted to evaluate the effectiveness of *Salix* bark extract for the treatment of low back pain. Patients received bark extract orally with 120 mg or 240 mg of salicin or placebo in 4-week. In the last week of treatment, 39% of patients were pain-free for the high dose, 21% for the low dose, and 6% for the placebo (Chrubasik et al., 2000). Another randomized placebo-controlled, double-blind study was conducted involving 78 patients with osteoarthritis. Patients received placebo or willow bark extract containing 240 mg salicin per day for 2 weeks. In the willow bark extract group, the WOMAC pain score was reduced by 14% after 2 weeks, compared with an increase of 2% in the placebo group. Therefore, *Salix* extract was well tolerated, with no adverse effects reported (Schmid et al., 2001). Patients (436) with osteoarthritis and rheumatic pain or back pain or both conditions received by 6-month mono or combination therapy with aqueous *Salix* extract, opioids, and NSAIDs. The results showed that in conjunction with the use of the *Salix* extract, the pain scores were between 33% and 44% of baseline. Drug interactions were not reported (Uehleke et al., 2013). Overall, the level of evidence is High for pain.

Safety. Oral administration of a *Salix alba* ethanol extract when administered to female rabbits and rats did not disrupt the estrous cycle, or inhibit ovulation or fertility and was not teratogenic or embryotoxic at a dose of 1.6 ml/kg (WHO, 2009). *Salix alba* ethanol extract (1.6 ml/kg, intragastric administration, in rats for 13 weeks) had no effect on kidney function, hematological parameters, cholesterol levels, or liver function. The results of the histological evaluation showed no pathological changes in the heart, brain, lungs, kidneys, bone, liver, mammary tissue, reproductive organs, intestines, or stomach (WHO, 2009). In mice (both sexes), *S. alba* ethanol extract presented a median lethal dose range of 28.0–42.0 ml/kg (WHO, 2009). In children can cause a risk of Reye’s syndrome, then it is not recommended (El-Radhi, 2018). Also, the use is not recommended in patients with hypersensitivity to other nonsteroidal anti-inﬂammatory drugs. People with asthma (sensibility to salicylates) due to severe reactions (acute bronchospasms) should avoid the use (WHO, 2009).

Specific warnings and precautions of use. In the case of treatment with anticoagulants, antacids, corticosteroids, or NSAIDs, the use of salix should be avoided (EMA, 2017d). Do not use it in people with gastrointestinal disorders and sensitivity to salicylic acid (BRASIL, 2011). Overall, due to the antiplatelet effect, in the context of COVID-19, the safety should be considered Medium.

Overall assessment. *Salix alba* profile and chemistry fit as an anti-inflammatory and antipyretic therapy in the context of upper respiratory affections. Therefore, this herbal medicine may be useful in the symptomatic relief of respiratory symptoms through exerting an anti-inflammatory and antipyretic effect. The clinical evidence is High. Due to its antiplatelet effects, this herbal medicine is considered presenting Medium safety in the COVID-19 context.

#### *Sambucus nigra* L. – Adoxaceae (Dried Flowers)

Indications in the context of respiratory conditions. *Sambucus nigra* is indicated for fever and inflammation of the respiratory tract (WHO, 2002; BRASIL, 2011). Other indications are to relieve cold and flu symptoms, to alleviate headaches, and as expectorant (EMA, 2018e; Torabian et al., 2019).

Chemical composition. Flavonoids, such as kaempferol, astragalin, quercetin, rutin, isoquercitrin, hyperoside; triterpenes (α- and β-amyrin, ursolic acid, oleanolic acid); sterols (β-sitosterol, campesterol, stigmasterol); phenolic acids and their corresponding glycosides (chlorogenic, ferulic, caffeic and *p*-coumaric acids); and essential oil (Sidor and Gramza-Michałowska, 2015).

Posology (based on traditional uses). 3–5 g of dried flowers in 25 ml as an infusion three times daily. Store in a well-closed container, protected from light (Bradley, 1992). 2–5 g dried flowers in 150 ml as an infusion, 2–3 times a day; 3–6 g dried flowers in 150 ml as a decoction, 2–3 times a day (EMA, 2018e).

Preclinical evidence. This herbal medicine has been experimentally proven for fever. The active constituents inhibit the biosynthesis of the inflammatory cytokines IL-1a, IL-1b, and tumor necrosis factor-α in human peripheral mononuclear cells *in vitro* (Yeşilada et al., 1997; Torabian et al., 2019). Several *in vitro* and *in vivo* experiments have shown the antiviral effects of extracts from elderberries, against *Influenza* virus (Serkedjieva et al., 1990; Zakay-Rones et al., 1995; Krawitz et al., 2011; Kinoshita et al., 2012; Porter and Bode, 2017).

Clinical evidence. *Sambucus nigra* preparations have been trialed clinically for common cold and flu. Two randomized studies with a standardized extract, involving patients presenting influenza symptoms and positive reaction for *Influenza* virus type A or B, showed the reduction of the symptoms after the second day of treatment, without significant adverse effects (Zakay-Rones et al., 1995; Zakay-Rones et al., 2004). In another randomized study with air travelers, the group using *S. nigra* presented less cold episodes than the placebo group (Tiralongo et al., 2016). A meta-analysis of randomized clinical studies led to the conclusion that *S. nigra* supplementation reduces upper respiratory symptoms of common cold and influenza (Hawkins et al., 2019). Overall, the level of evidence is High for cold and flu.

Safety. No information available on general precautions or concerning drug interactions; non-teratogenic effects in pregnancy or pediatric use (EMA, 2017c). Overall, safety is High.

Specific warnings and precautions of use. None.

Overall assessment. *Sambucus nigra* is traditionally used as cold therapy in the context of upper respiratory conditions and could be useful in the relief of respiratory symptoms through exerting anti-inflammatory and antipyretic effects. The clinical evidence is High, and this herbal medicine is considered presenting High safety.

#### *Scutellaria baicalensis* Georgi – Lamiaceae (Roots)

Indications in the context of respiratory conditions. *Scutellaria baicalensis* is indicated for fever (WHO, 2007). Other related indications are as antiviral and to relieve bronchitis symptoms (WHO, 2007; Zhao et al., 2019).

Chemical composition. Flavonoids such as wagonin, baicalcin, and baicalin are the main components (Zobel et al., 1991; Kovács et al., 2004; Reichling and Galati, 2004; Abdollahi Fard, 2012); essential oil (Fukuhara et al., 1987); terpenoids and steroids (e.g., β-caryophyllene, scutebaicalin, β-sitosterol) (Hussein et al., 1996).

Posology (based on traditional uses). 1-2 g of dried roots in 150 ml of boiling water, as an infusion, three times daily (Barnes et al., 2012). 3-9 g of dried roots as infusion or decoction (WHO, 2007).

Preclinical evidence. *Scutellaria baicalensis* has been not experimentally proven for symptoms of respiratory disease. Other related experimental effects include anti-inflammatory. The anti-inflammatory activity was evaluated by *in vitro* and *in vivo* experiments. Extracts inhibited the production of NO, interleukin (IL)-3, IL-6, IL-10, IL-12p40, IL-17, interferon-inducible protein (IP)-10, keratinocyte-derived chemokine, and vascular endothelial growth factor (VEGF) in LPS-induced RAW 264.7 cells (Yoon et al., 2009). Moreover, the aqueous extract of *S. baicalensis* suppressed LP-induced COX-2 protein expression *in vitro*, as well as presented analgesic and anti-inflammatory activities *in vivo* (Lee et al., 2007).

Clinical evidence. This herbal medicine has been not trialed clinically for the following symptom: bronchitis, cough, fever, flu, cold. Overall, the clinical evidence is Low.

Safety. Some stomach discomfort and diarrhea were observed in patients taking preparation of *S. baicalensis* (WHO, 2007). In a randomized, double-blind trial, involving 72 healthy subjects, baicalein (100–2,800 mg) was safe and did not present severe adverse effects (Li et al., 2014). There are reports of baicalein being able to stimulate the ACE2 activity, though (ANSES, 2020). Therefore, safety is Medium.

Special warnings and precautions of use. *Scutellaria baicalensis* should not be used in people with spleen and stomach deficiency (Dinehart and Henry, 2005).

Overall assessment. *Scutellaria baicalensis* may be useful in the relief of respiratory symptoms through the anti-inflammatory activity. The clinical evidence is Low. This herbal medicine is considered presenting Medium safety.

#### *Silybum marianum* L. Gaertn. - Asteraceae (Fruits)

Indications in the context of respiratory conditions. *Silybum marianum* is indicated for symptoms of respiratory disease, namely fever, and catarrh (WHO, 2002).

Chemical composition. Flavonolignans such as silymarin, silybin, isosilybin and taxifolin (1.5–3.0%) are the main components reported for *S. marianum* (Wynn, 1999; Porwal et al., 2019); flavonoids (quercetin derivatives); phenolic acids (chlorogenic, caffeic and ferulic acids) (Lucini et al., 2016; Valková et al., 2020).

Posology (based on traditional uses). It is preferable to use a commercial preparation with a defined composition and an adequate dose.

Preclinical evidence. *Silybum marianum* has not been experimentally proven for symptoms of respiratory disease. Silybin, the main flavonolignan from the seed of *S. marianum*, has demonstrated an anti-inflammatory activity by inhibiting the spontaneous and LPS-stimulated NF-kB activation as well as the production of inflammatory cytokines (Corchete, 2008; Giorgi et al., 2012).

Clinical evidence. *Silybum marianum* has not been trialed clinically for respiratory diseases. Overall, the clinical evidence is Low.

Safety. At high doses, mild laxative effect, as well as mild allergic reactions, have been reported (Blumenthal, 2003; Lucini et al., 2016), besides dry mouth, nausea, upset stomach, gastric irritation or headache (EMA, 2016d). A preparation containing 7%–8% silymarin appeared to be safe for up to 41 months of use (Corchete, 2008). Overall, safety is High.

Specific warnings and precautions of use. None.

Overall assessment. Although *S. marianum* is not clinically proven to provide symptomatic relief of flu symptoms, it could be useful in the relief of respiratory symptoms by exerting a systemic anti-inflammatory effect. The clinical evidence is Low. This herbal medicine is considered presenting High safety.

#### *Thymus vulgaris* L. - Lamiaceae (Aerial Parts, Leaves)

Indications in the context of respiratory conditions. *Thymus vulgaris* is indicated for productive cough associated with cold, laryngitis, and tonsilitis (WHO, 1999; CHILE, 2010; EMA, 2013c).

Chemical composition. Essential oil (thymol as the major component); phenolic acids (rosmarinic, ferulic, syringic, coumaric acids), tannins (gallic and protocatechuic acids), flavonoids (luteolin, apigenin, cirsilenol, thymonin, and derivatives, besides others) (Van Den Broucke and Lemli, 1983; Kuete, 2017; Salehi et al., 2019a).

Posology (based on traditional uses). 1–2 g of dried leaves in 150 ml, as an infusion, 3–4 times daily (EMA, 2013c).

Preclinical evidence. *Thymus vulgaris* has not been experimentally proven for symptoms of respiratory disease. However, the myorelaxant effect of ethanolic extract and flavonoids from *T. vulgaris* was evaluated in isolated guinea-pig trachea contracted by acetylcholine. The extract promoted relaxation, similar to DL-isoprenaline (Van Den Broucke and Lemli, 1981). The most active flavonoids were cirsilineol, thymonin, and 8-methylcirsilineol, acting as non-competitive and non-specific Ca^2+^ antagonists (Van Den Broucke and Lemli, 1983). An evaluation of *T. vulgaris* essential oil in rats, through classic methods, showed that the essential oil presents anti-inflammatory activity. Additional experiments showed that carvacrol plays an important role in the activity, with anti-edematogenic and anti-chemotactic action. The inhibition of chemotaxis was also proven by *in vitro* experiments. On the other hand, thymol promoted the release of histamine acting as an irritative agent (Fachini-Queiroz et al., 2012). The hydroethanolic extract of *T. vulgaris* also presents myorelaxant activity *ex vivo* (Meister et al., 1999; Boskabady et al., 2006b). An aqueous extract of *T. vulgaris* presented *in vitro* activity against *Herpes simplex* virus type 1 (HSV-1), type 2 (HSV-2), and HSV-1 acyclovir-resistant (Nolkemper et al., 2006).

Clinical evidence. This herbal medicine has been not trialed clinically, as monodrug, for respiratory disease. Clinical evaluation of thyme has been carried out as an association with another herbal drug, such as *P. vulgaris*. Overall, the clinical evidence is Low.

Safety. In traditional doses, there is no report about the toxicity of *T. vulgaris*. However, high doses (around 10 g) are considered unsafe due to thymol (Basch et al., 2004). Overall, safety is High.

Specific warnings and precautions of use. The use is contraindicated for hypersensitivity to the active substance. Children under 30 months of age should not use this plant due to thymol containing preparations, which can induce laryngospasm (WHO, 1999; EMA, 2013c).

Overall assessment. Although *T. vulgaris* is not clinically proven to provide symptomatic relief of flu symptoms, it may be useful in the relief of respiratory symptoms through spasmolytic and anti-inflammatory effects. The clinical evidence is Low. This herbal medicine is considered presenting High safety.

#### *Tilia cordata* Mill. – Malvaceae (Flowers)

Indications in the context of respiratory conditions. *Tilia cordata* is indicated for symptoms of the common cold (CHILE, 2010; EMA, 2012c).

Chemical composition. Essential oil (main components are alkanes, such as 6,10,14-trimethyl-2-pentadecanone and tricosane, besides linalool, E-anethole) (Fitsiou et al., 2007; Kowalski et al., 2017); flavonoids (e.g., quercetin, kaempferol, and derivatives; tiliroside), anthocyanins (Negri et al., 2013; Fawzy et al., 2018).

Posology. 1.5 g in 150 ml of boiling water, as an infusion, 2–4 times daily (CHILE, 2010; EMA, 2012c)

Preclinical evidence. This herbal medicine has not been experimentally proven for the symptoms of respiratory disease. However, procyanidins present in small flowers were evaluated about an inflammatory response in human neutrophils in the concentration ranging from 5 to 20 μM. All compounds were able to decrease ROS production from f-MLP-stimulated neutrophils. Most compounds were able to inhibit LPS-induced IL-8 release. Some trimeric and tetrameric derivatives were also able to decrease the production of MIP-1β. A hydromethanolic extract presented anti-inflammatory and antinociceptive activities in rats, in a similar way than indomethacin and acetylsalicylic acid, respectively (Fawzy et al., 2018).

Clinical evidence. This herbal medicine has been not trialed clinically for respiratory diseases. Overall, the level of evidence is Low.

Safety. Overall safety is High, as there are not reports to infer that the preparations from the plant may interfere with the disease.

Specific warnings and precautions of use. Flavonoids present in *T. cordata* exert an anxiolytic effect and can, potentially, interact with serotonergic drugs (Noguerón-Merino et al., 2015).

Overall assessment. *Tilia cordata* is not clinically proven to provide symptomatic relief of flu symptoms. However, this herbal medicine may be useful in the relief of respiratory symptoms through an anti-inflammatory effect. The clinical evidence is Low. This herbal medicine is considered presenting High safety.

#### *Zingiber officinale* Roscoe - Zingiberaceae (Rhizome)

Indications in the context of respiratory conditions. *Zingiber officinale* is indicated for common cold and cough (COLOMBIA, 2008). Its use as anti-asthma (WHO, 1999) and expectorant (BRASIL, 2011) have also been reported.

Chemical composition. Zingerone, shogaols, gingerols, paradols, wikstromol, and carinol (Idris et al., 2019). Total phenolic of 1.02 ± 0.03 mg gallic acid equivalent/L of infusion (Mesquita et al., 2018).

Posology (based on traditional uses). 1 g of dried rhizoma in 150 ml, 3–4 times daily (BRASIL, 2011; EMA, 2011b).

Preclinical evidence. This herbal medicine has been experimentally proven for fever (Ueki et al., 2008; Akbar, 2020). It is described in several preclinical studies report its anti-inflammatory, antipyretic and analgesic properties (Mascolo et al., 1989; El-Abhar et al., 2008; Sepahvand et al., 2010; Ahmed et al., 2011; Darvishzadeh-Mahani et al., 2012; Hsiang et al., 2013; Rashidian et al., 2014). The anti-inflammatory effect of ginger is well established as *in vivo* as *in vitro* models (Thomson et al., 2002; Ali et al., 2008). 6-Shogaol interferes with the inflammatory cascade, inhibits COX, and the prostaglandin release (Rehman et al., 2011). Moreover, 6-gingerol and 6-shogaol present anti-platelet aggregation activity *in vitro* (Liao et al., 2012).

Clinical evidence. This herbal medicine has been trialed clinically for respiratory diseases. In a randomized study, 32 patients with acute respiratory distress syndrome (ARDS) received an enteral diet enriched with ginger or placebo, through a nasogastric tube, during 21 days. On day 5, the patients that received ginger presented lower serum levels of IL-1, IL6, and TNFa, while the level of RBC glutathione presented higher in comparison with the placebo group. Moreover, a significant improvement in oxygenation was observed in the ginger group. The same results were observed on day 10. The authors described that there was a significant difference in the duration of mechanical ventilation and in the time expended in the intensive care unit. However, barotrauma, organ failure, and mortality between ginger and placebo groups were similar (Vahdat Shariatpanahi et al., 2013). Overall, the level of clinical evidence is Medium.

Safety. Although regulatory authorities recommend avoiding the use in pregnancy and lactation (EMA, 2011b), ginger use during pregnancy was reported as safe in Canadian, Australian and Norwegian women (Portnoi et al., 2003; Willetts et al., 2003; Westfall, 2004; Heitmann et al., 2013). Clinical studies found ginger as effective and safe to prevent pregnancy-associated nausea and vomiting (Borrelli et al., 2005; Ding et al., 2013). In human liver microsomes, the hydroethanolic extract inhibited CYP2C19 and CYP2D6 (Kim et al., 2012; Gorman et al., 2013).

Specific warnings and precautions of use. Stomach upset, eructation, dyspepsia, and nausea have been reported (EMA, 2011b). High doses (12–14 g) of ginger may increase the effects of anticoagulant therapy (Thomson et al., 2002; BRASIL, 2011; BRASIL, 2016). Studies showed the effect of ginger on cytochrome P450 enzyme-mediated drug metabolism, which can cause interference on the outcome of the treatment of some conventional drugs. Ginger stimulated levels of CYP450 and cytochrome b5 in rats (Sambaiah and Srinivasan, 1989). The use is contraindicated for people with gallstones, gastric irritation, and high blood pressure (BRASIL, 2011). Overall, safety is Medium due to the potential antiplatelet activity.

Overall assessment. *Zingiber officinale* profile and chemistry fit as an anti-inflammatory therapy in the context of upper respiratory affections. Therefore, this herbal medicine may be useful in the relief of respiratory symptoms through exerting an anti-inflammatory effect. The clinical evidence is Medium. This herbal medicine is considered presenting Medium safety.

### Herbal Remedies Used to Relieve Symptoms Related to Respiratory Conditions Not Listed By International Monographs

Some species, although without the support of official documents, have been widely used to relieve common cold symptoms, usually as homemade preparations. Some of them are analyzed here, in the COVID-19 context.

#### *Citrus limon* (L.) Burm. f. – Rutaceae (Fruit)

Indications in the context of respiratory conditions. *Citrus limon* has been used to relieve cough, and as expectorant in bronchitis (Papp et al., 2011; Klimek-Szczykutowicz et al., 2020); and as anti-inflammatory (Parhiz et al., 2015), sore throat (Balogun and Ashafa, 2019).

Chemical composition. Essential oil (limonene and α-terpineol as major components) (Mahalwal and Ali, 2003); flavonoids (e.g., hesperidin, hesperetin, naringenin) (Abad-García et al., 2012; Parhiz et al., 2015); phenolic acids (e.g., *p*-cumaric, ferulic, quinic acids and derivatives) (Abad-García et al., 2012), coumarins and furanocoumarins; vitamin C (Klimek-Szczykutowicz et al., 2020).

Posology. Slices of whole fruit or fresh pericarp, as a decoction, 2–3 times daily; expressed juice (Panizza et al., 2012).

Preclinical evidence. This herbal medicine has not been experimentally proven for respiratory disease.

Clinical evidence. This species has not been trialed clinically for respiratory disease. Overall the clinical evidence is Low.

Safety. *Citrus limon* is an edible fruit, therefore, considered safe, although there may be a risk of minor burns on the skin if in contact with sun exposure, due to the presence of furanocoumarins (Palazzolo et al., 2013). Overall safety is High.

Specific warnings and precautions of use. *Citrus* flavonoids can exert severe interaction with drugs metabolized by inhibition of CYP3A4, CYP2C9 (Bailey et al., 1998; Mallhi et al., 2015). *Citrus limon* presents anti-platelet activity. Therefore, the concomitant use with anti-platelet drugs should be avoided.

Overall assessment. *Citrus limon* can be useful in the relief of respiratory symptoms, especially cough and sore throat. The clinical evidence is Low. Due to its antiplatelet effects, this herbal medicine is considered presenting Medium safety in the COVID-19 context.

#### *Culcitium canescens* H&B. – Asteraceae (Leaves and Roots)

Indications in the context of respiratory conditions. In Peru and adjoining countries, *Culcitium canescens* is used to relieve cough and fever (Okuyama et al., 1994). Another related indication includes asthma (D’Agostino et al., 1995; Ramirez et al., 2020)

Chemical composition. Furanoeremophilanes (e.g., cacalonol, cacalohatine, dehydrocacalohastine) (Abdo et al., 1992; Okuyama et al., 1994); phenolic compounds (e.g., kaempferol and derivatives, quercetin and derivatives, caffeic acid) (D’Agostino et al., 1995); terpenes and steroids (spatulenol, lupeol, damaradienone) (Ramirez et al., 2020).

Posology (based on traditional uses). 1–2 g of leaves in 150 ml, as an infusion, up to three times daily (Ramirez et al., 2020).

Preclinical evidence. *Culcitium canescens* has not been experimentally proven for respiratory disease.

Clinical evidence. This species has not been trialed clinically for respiratory disease. Overall the clinical evidence is Low.

Safety. Little or anything is known about the toxicity of this plant (Ramirez et al., 2020).

Specific warnings and precautions of use. None.

Overall assessment. Although *Culcitium canescens* profile and chemistry fit as an anti-inflammatory therapy in the context of upper respiratory affections, and preparations are traditionally used, the lack of knowledge about this species does not allow a clear recommendation for the relief of early symptoms of COVID-19. The clinical evidence is Low.

#### *Laurus nobilis* L. – Lauraceae (Berries/Leaves)

Indications in the context of respiratory conditions. *Laurus nobilis* is used to treat respiratory infections (Chevallier, 1996; Ross, 2001).

Chemical composition. Berries: essential oil (β-ocimene and 1,8-cineole as the main components); sterols (e.g., β-sitosterol); flavonoids (cyanidin 3-O-glucoside and cyanidin 3-O-rutinoside, as the major anthocyanins) (Kilic et al., 2004; Beis and Dunford, 2006; Abu-Dahab et al., 2014). Leaves: essential oil (1,8-cineole and *a*-terpinyl acetate as the major compounds) (Fidan et al., 2019). Further phytochemical investigations of laurel leaves led to the isolation of sesquiterpene lactones, alkaloids, glycosylated flavonoids, and monoterpene and germacrane alcohols (Dall’Acqua et al., 2009).

Posology (based on traditional uses). The only dosage information on *L. nobilis* is given by the American Pharmaceutical Association: 1–2 tablespoons leaf/cup water and 3 times/day or 1–2 drops of essential oil added to honey, or tea (Duke et al., 2002).

Preclinical evidence. This species has not been experimentally proven for respiratory disease. The closest experiment is an *in vitro* study examining SARS-CoV and the effect of several essential oils. The authors reported that a distilled oil extracted from *Laurus nobilis* berries was an effective virucidal against SARS-CoV. This essential oil also contained eremanthin and dehydrocostus lactone as minor constituents at 3.65% and 7.57%, respectively. These compounds are somewhat unusual in essential oils, but at least one *in vitro* study found that dehydrocostus lactone had activity against the hepatitis B virus, an enveloped DNA virus (Loizzo et al., 2008). The essential oil of *L. nobilis* has also been evaluated for its antinociceptive and anti-inﬂammatory activities in mice and rats. The essential oil exhibited a signiﬁcant analgesic effect in tail-ﬂick and formalin tests, a dose-dependent anti-inﬂammatory effect in formalin-induced edema, and a moderate sedative effect at the anti-inﬂammatory doses (Sayyah et al., 2003).

Clinical evidence. This species has not been trialed clinically for respiratory disease. Overall, the clinical evidence is Low.

Safety. There is not enough reliable information about the safety of taking *L. nobilis* leaves and berries. As *L. nobilis* has been used as food since ancient times, overall, it can be considered safe in the recommended doses. Therefore, safety is High.

Specific warnings and precautions of use. None.

Overall assessment. *Laurus nobilis* profile and chemistry fit as an anti-inflammatory therapy in the context of upper respiratory affections. Therefore, this herbal medicine could be useful in the relief of respiratory symptoms through exerting anti-viral and anti-inflammatory effects on the respiratory tract. The clinical evidence is Low. This herbal medicine is considered presenting High safety.

#### *Lippia graveolens* Kunth. – Verbenaceae (Leaves, Aerial Parts)

Indications in the context of respiratory conditions. *Lippia graveolens* is used to treat symptoms of respiratory diseases, and as anti-inflammatory (Rastrelli et al., 1998).

Chemical composition. Iridoid and secoiridoid glucosides and ester derivatives (Rastrelli et al., 1998); essential oil (thymol, limonene, carvacrol, as the major components) (Compadre et al., 1987; Uribe-Hernández et al., 1992); lipids (esterols) and flavonoids (e.g., pinocembrin, luteolin, apigenin, quercetin and derivatives) (Lin et al., 2007; Arias et al., 2020); rosmarinic acid (Compadre et al., 1987).

Posology (based on traditional uses). 1–2 g of dried leaves or stem in 150 ml of water (Dominguez et al., 1989).

Preclinical evidence. This species has not been experimentally proven for respiratory disease. Flavonoid and terpenoid fractions from *Lippia* genus have demonstrated a significant inhibitory effect on ROS and NO production and mitochondrial activity in LPS-induced inflammation in RAW 264.7 macrophage cells. Additionally, these fractions exhibited non-selective inhibitions against the activity of the cyclooxygenases COX-1 and COX-2 (Leyva-López et al., 2016).

Clinical evidence. This species has not been trialed clinically for respiratory disease. Overall, the clinical evidence is Low.

Safety. In the United States, the regulatory status “generally recognized as safe” has been accorded to Mexican oregano (GRAS 2827). Therefore, safety is High.

Specific warnings and precautions of use. None.

Overall assessment. *Lippia graveolens* profile and chemistry fit as an anti-inflammatory therapy in the context of upper respiratory affections. Therefore, this herbal medicine could be useful in the relief of respiratory symptoms through exerting an anti-inflammatory effect. The clinical evidence is Low. This herbal medicine is considered presenting High safety.

#### *Nigella sativa* L. - Ranunculaceae (Seeds)

Indications in the context of respiratory conditions. Black seeds are globally known as a spice and as such as a food item. In Arabic medicine, they have very high status as an herbal medicine used for a wide range of diseases. In the context of COVID-19, the relevant uses are for asthma and in the more general management of inflammatory conditions. In many Arabic countries, black seed is used for asthma and cough, among many other uses, especially for gastrointestinal diseases (like abdominal pain, stomach ache, colic), rheumatism, skin diseases (Lebling and Pepperdine, 2006).

Chemical composition. Relevant secondary metabolites in the seed include large quantities of fixed oil (30%–45%; especially linoleic and oleic acids), various triterpenoids, including saponins; essential oil, with a relatively high concentration of thymoquinone (2-isopropyl-5-methyl-1,4-benzoquinone), which is also a key therapeutically relevant of the fixed oil, where it can be found in concentrations varying from 3.5 to 8.7 mg/g of fixed oil (Lutterodt et al., 2010). Thymoquinone is not found in relevant concentrations in aqueous preparations.

Posology. Capsules containing fatty oil with a defined amount of thymoquinone are likely to be the best option. Herbal teas and other hydrophilic preparations seem less likely to have benefits, and potentially the essential oil may offer some relief.

Preclinical evidence. In rats, a chemically not well characterized 50% ethanol extract (1:4) of chopped *N. sativa* seeds (extraction at room temperature for 72 h) showed ameliorating effects on lung inflammation and oxidative stress induced by lipopolysaccharide. In general, many study focus on the effects of *N. sativa* preparations (Yimer et al., 2019), but mostly these are of highly inadequate scientific quality.

Clinical evidence. Some small studies demonstrate clinical benefits in asthma patients. In a randomized, double-blind, placebo-controlled trial supplementation of standard therapy with soft gel capsules of cold-pressed *N. sativa* oil (0.7% thymoquinone) improved asthma control with a trend in pulmonary function improvement. Two of the five subscores (impact of asthma on work, school, or at home and shortness of breath and the overall score) showed a significant improvement. The other three were not significantly improved. At the same time, a remarkable normalization of blood eosinophilia count was observed (Koshak et al., 2017). The release of IL-2, IL-6, and PGE2 in T-lymphocytes, as well as IL-6 and PGE2 in monocytes, were suppressed. Overall, the clinical evidence is Medium for asthma.

Safety. As a commonly used food item, it can be considered to be intrinsically safe. However, this does not apply to the lipophilic extracts discussed above. Here further evidence is essential. Insufficient evidence is available in the context of uses as an adjuvant medication (Nguyen et al., 2019). Overall, safety is High.

Specific warnings and precautions of use. Again, there is no evidence for specific therapeutic benefits, and it is important to communicate this to potential users. There is insufficient evidence for use during pregnancy and while breastfeeding.

Overall assessment. Black seed may be useful in the relief of respiratory symptoms mainly associated with the severe cough experienced, and it may reduce inflammatory parameters. The clinical evidence is very limited, focusing on asthma. A particular concern, in this case, are the many well-intended but very low-quality studies and the broad range of claims they try to support, making any assessment problematic. The clinical evidence is Medium. This herbal medicine is considered presenting High safety.

#### *Plectranthus amboinicus* Lour. – Lamiaceae (Leaves)

Indications in the context of respiratory conditions. *Plectranthus amboinicus* has been used in asthma and to relieve cold, headache, and fever (Menéndez Castillo and Pavón González, 1999; Duke et al., 2002).

Chemical composition. Essential oil (thymol and carvacrol as the major components) (Pino et al., 1990; Arumugam et al., 2016); phenolic compounds (e.g., rosmarinic, caffeic, gallic and *p*-coumaric acids; quercetin derivatives; luteolin) (Brieskorn and Riedel, 1977b; Arumugam et al., 2016), terpenoids and steroids (e.g., β-sitosterol, α-amyrin, stigmasterol) (Brieskorn and Riedel, 1977a; Risch et al., 2012).

Posology (based on traditional uses). 3–5 g of fresh leaves in 150 ml, as an infusion, up to three times daily (Albornoz, 1993; Matos et al., 2001).

Preclinical evidence. This herbal medicine has not been experimentally proven for respiratory disease. Aqueous and ethanolic extracts of *P. amboinicus* present *in vitro* anti-inflammatory activity (Devi and Periyanayagam, 2009; Ravikumar et al., 2009; Janakiraman and Somasundaram, 2014). The active constituents of *P. amboinicus* present inhibitory effects on pro-inflammatory cytokines TNF-α, IL-6, and IL-1β (Yashaswini and Vasundhara, 2011).

Clinical evidence. This species has not been trialed clinically for respiratory disease. Overall, the clinical evidence is Low.

Safety. No toxicity reported at the indicated dose (Albornoz, 1993; Matos et al., 2001). Overall, safety is High.

Specific warnings and precautions of use. The use is contraindicated for hypersensitivity to the active substance. Children, under 30 months of age, should not use this plant due to thymol containing preparations, can induce laryngospasm (see *Thymus vulgaris*).

Overall assessment. *Plectranthus amboinicus* profile and chemistry fit as an anti-inflammatory therapy in the context of upper respiratory affections and could be useful in the relief of respiratory disease through exerting an anti-inflammatory effect on the respiratory tract and in the treatment of fever. The clinical evidence is Low, but this herbal medicine is considered presenting High safety.

### Discussion

#### General Discussion About the Position of International Health Organizations, National Authorities, and Professional Bodies on the Use of Herbal Medicines Within the Context of COVID-19 Disease

The use of herbal medicines/food supplements to prevent, treat, mitigate, diagnose, or cure coronavirus disease 2019 has not been consistently addressed at a global level. China has been actively exploring how to integrate traditional Chinese medicine (TCM) into western therapy since the SARS outbreak in 2003. Based on clinical results, the General Office of the National Health, and the Office of the State Administration of Traditional Chinese Medicine encouraged the integration of herbal TCM and Western medicine in the treatment of respiratory complications in Coronavirus infections with different prescriptions recommended in different stages of disease (Leung, 2007). After SARS-CoV-2 disease outbreak, a number of clinical trials with Traditional Herbal Medicines (THM) have been added to the body of evidence with some favorable findings when compared to standard antiviral therapy (Langeder et al., 2020; Li et al., 2020; Luo et al., 2020; Rastogi et al., 2020).

Therefore, it is not surprising that thousands of TCM practitioners across China have been working along with regular medical doctors to control the COVID-19 disease. The clinical data generated from tens of thousands of confirmed cases are being subjected to meta-analyses to ascertain their actual effectiveness (Li et al., 2020). In the meantime, the Diagnosis and Treatment Protocol of COVID-19 by the National Health Commission has already advised to integrate TCM in the treatment of COVID-19 patients to effectively relieve symptoms such as fever, cough, sore throat, myalgia, and fatigue, shorten the course of the disease, reduce the probability of life-threatening complications (Xu et al., 2020).

In India, the Ayurveda Practitioners are following suit by working on setting clear protocols with a rationale consistent with their millenary system. The first line of defense recommended to early-stage patients consists of the following herbal medicines: *Tinospora cordifolia* (Willd.) Myers (Menispermaceae), *Zingiber officinale* Roscoe (Zingiberaceae), *Curcuma longa* L. (Zingiberaceae), *Ocimum sanctum* L. (Lamiaceae), *Glycyrrhiza glabra* L. (Fabaceae), *Adhatoda vasica* Ness (Acanthaceae), (Acanthaceae), *Andrographis paniculata* (Burm.f.) (Acanthaceae)*, Swertia chirata* Buch.-Ham. ex Wall. (Gentianaceae), *Moringa oleifera* Lam (Moringaceae), Triphala [a mixture of the dried fruits of *Emblica officinalis* Gaertn. (Phyllanthaceae)*, Terminalia bellirica* (Gaertn.) Roxb. (Combretaceae), and *Terminalia chebula* Retz.(Combretaceae)] and Trikatu [a complementary formula to Triphala, including *Piper nigrum* L. (Piperaceae)*, Piper longum* L. (Piperaceae), and *Z. officinale* Roscoe] (Rastogi et al., 2020). Clinical settings and regulations of research on COVID-19 through Ayurveda, Unani, Siddha, and Homeopathy systems have already been published by the Indian Ministry of Ayush (INDIA, 2020).

However, in Western medicine, we are witnessing quite the opposite. Not only integration of herbal medicine is “out of the question” from an emergency clinical point of view, but authorities are also reluctant - if not discouraging - the self-prescription of any products other than paracetamol in early stages of the disease. The media trying to get answers about the benefits of herbal medicines turn to medical experts who usually caution against the use of herbal medicines in order to avoid any liabilities. Oftentimes, dismissing the potential of herbal medicines to relieve early COVID-19 symptoms is just an uncritical and legalist approach. An international initiative from Brazilian Academic Consortium for Integrative Health - CABSIn, TCIM Americas Network, and the Latin-American and Caribbean Center on Health Sciences Information (BIREME/PAHO/WHO) is systematizing the available evidence about Traditional, Complementary and Integrative Medicine (including herbal medicines) potentially useful in the COVID-19 pandemic (Portella et al., 2020).

Traditional herbal medicines are poorly or not regulated as licensed/registered medicines in quite a few countries, including the USA, and therefore are considered “food,” precluding any high-level discussion on their potential contributions to human health. Consequently, the U.S. Food and Drug Administration (FDA) considers fraudulent to sell any herbal products with attached claims and has sent warning letters to companies offering herbal products such as teas, essential oils, and tinctures claiming to prevent, treat or cure the symptoms of COVID-19. It is also encouraging the public to report the unlawful sales of medicinal products on the Internet (FDA, 2020). The North American public is receiving advice on the use of herbal products from dedicated academic groups. One of them is the Andrew Weil Center for Integrative Medicine which recommends polyphenol-rich plants (chamomille, Chinese skullcap, licorice, onions, apples, tomatoes, oranges, nuts, berries, parsley, celery, turmeric root, and green tea) to reduce the risk of infection (Alschuler et al., 2020), but no detailed assessment is available.

In Europe, Traditional Herbal Medicine Products (THMP) are regulated by the EMA as OTC medicinal products for the relief of mild, self-limiting conditions, including flu symptoms and cough. Surprisingly, there is not any overarching official guidance with respect to the use of THMP in the context of the pandemic. In principle, THMP would not qualify as therapy for COVID-19 symptoms as it is considered a severe life-threatening condition. However, this disease is self-limiting in most of the patients and - in the absence of a positive result in a test – COVID-19 patients are just ‘flu’ patients and, as such, could self-medicate with THMP. Several herbal drugs here reviewed have been registered as THMP. EMA stresses in its guidelines to healthcare professionals to report all “*suspected side effects your patients experience while infected, regardless of the medicines intended to treat the disease or pre-existing conditions. Suspected side effects should be reported even if the medicine is not authorized for use in COVID-19. When reporting a suspected side effect in a patient, you should tell us of any other medicines being taken around the same time, including non-prescription medicines, herbal remedies, or contraceptives*” (EMA, 2020).

We have witnessed how, in different countries, the authorities have developed an array of tools both to inform and enforce guidelines: Italian police are acting at retail commerce level by seizing on the spot any products sold under fraudulent claims; the Brazilian Ministry of Health is fighting back any misleading claims with a dedicated channel in WhatsApp (BRASIL, 2020). The case of France is unique in that its health authorities have provided an extensive and detailed analysis of the potential interactions of natural products with either the disease or its clinical treatment (ANSES, 2020). Finally, many other countries lack the resources to either reach out to the public in this specific matter or enforce any significant actions regarding to the sale of such products.

The WHO regional office for Africa has issued a statement supporting scientifically-driven traditional medicine as “a valid approach towards the treatment of the virus, as long as their efficacy and safety are proven through rigorous clinical trials” (WHO, 2020b), in reaction to a self-proclaimed “COVID-19 herbal cure” promoted at high governmental levels in Madagascar under the name COVID Organics (CVO) (BBC, 2020; WHO, 2020b).

The potential risks and public impact associated with any advice on this disease are huge, and understandably; so, Herbalists associations are more cautious than usual in promoting herbal medicines in this context. In the UK, the NMHI just encourages its members to “integrate the herbal advice they provide with any conventional treatment or management strategies that are appropriate for their patients, and to observe the current infection control and management guidelines formulated by the Department of Health” (NMHI, 2020); and the College of Medicine actively recommends dietary interventions such as boosting immunity against coronavirus by ‘turning’ to antioxidants and polyphenol-rich food (Dixon, 2020).

### General Discussion About the Efficacy of Reference Drugs and Herbal Medicines in the Relief of Flu Symptoms in the Context of COVID-19

Despite the massive use of codeine, paracetamol, and ibuprofen for the relief of respiratory diseases, the clinical body of evidence supporting their use in flu is very low. These drugs are mostly used based on medical experience and clinicians are familiar with their benefits/risks profiles. These drugs have very common toxicities and their adverse effects are potentially dangerous in the context of COVID-19 (Jóźwiak-Bebenista and Nowak, 2014; Drugs.com, 2020a; Drugs.com, 2020b).

It is surprising the lack of clinical trials to substantiate the use of both paracetamol and ibuprofen in the treatment of cold/flu symptoms. As noted in previous sections, clinical evidence is somewhat higher for ibuprofen than for paracetamol.

### General Discussion About the Safety of Reference Drugs and Herbal Medicines in the Relief of Flu Symptoms in the Context of COVID-19

There is quite a consensus in that the patient may safely choose to use herbal medicines and food supplements in the prevention of COVID-19. In line with EMA, we advise the patients to keep a diary of the medication (herbal or not) to inform medical doctors and other health care professionals in case of hospitalization worsening of the disease.

Two types of interactions are important in COVID-19: disease-herbal medicine and herbal medicine-drug interactions. There is a complicated relationship between the immune system and viral infections: infection is favored by a weak immune response, whilst complications arise from an ‘overreaction’ of the immune system known as “cytokines storm”. As a result, health authorities discourage any therapies modulating the function of immune cells in any of both directions -including anti-inflammatory drugs- and reserve those to clinical management of advanced stages of COVID-19.

To complicate matters further, a significant fraction of COVID-19 patients in advance stages may require emergency treatment consisting of mechanical ventilation under general anesthesia. It is standard protocol for anesthetists to advise against taking herbal supplements at least 2 to 3 weeks before surgery or other procedures requiring anesthesia, due to potential pharmacodynamic and pharmacokinetic interactions with these procedures (Levy et al., 2017).

As a result of all the above, the use of any herbal-based products has been strongly discouraged by medical authorities, even for typical symptoms of the early stages of COVID-19. Patients are asked to rest, take paracetamol - if fever is dangerous only - and plenty of water. Such “Keep calm and carry on” clinical advice is not realistic and causes dissatisfaction in patients that are not ready to just ‘sit and suffer’ with the added distress of the potentially lethal complications. It is all human that they will try whatever is offered from alternative medicinal systems to minimize both symptoms and viral load.

Interestingly, there is seldom any mention to anti-tussive medication, one of the most characteristic symptoms of the COVID-19 disease, which chiefly contributes to its spread. Available cough medicines range from OTC products such as simple linctus (usually containing citric acid in a water-glycerol base) and THMP (in Europe) to pharmacy/prescription-only opioids such as dextromethorphan or codeine.

#### Potential Drug-COVID-19 Interactions

Early clinical data on the evolution of COVID-19 patients pointed out to a deleterious influence of ibuprofen and ketoprofen even taken as short as 2–4 days during the onset of the infection (ANSES, 2020), and prompted French authorities to discourage public to use NSAIDs other than paracetamol. Later data did not clearly substantiate the link between NSAIDs and worsening of the symptoms, so EMA released a communication clarifying this point and calling for rational use of NSAIDs ‘at the lowest effective dose for the shortest possible period’ (EMA, 2020).

The basis for lower use of NSAIDs has been substantiated on two lines of thinking: (a) they non-specifically impair the immune response thus potentially facilitating the viral infection; (b) some NSAIDs such as ibuprofen, potentially increase the expression of proteins necessary for the entry of the virus into the cell. The later includes proteins vimentin (streptococcus) and ACE-2 (coronavirus).

Surprisingly, paracetamol counts among its many potential adverse effects “very common incidence” (1% to 10%) of dyspnea, abnormal breath sounds, pulmonary edema, hypoxia, pleural effusion, stridor, wheezing, and coughing.

#### Potential Herbal-COVID-19 Interactions

##### Herbal Medicines Acting as NSAIDs and/or Immunomodulators

The French authorities have issued a detailed document warning against all herbal medicines with anti-inflammatory and immunomodulatory activities and discouraging their liberal use if flu symptoms appear, whilst the COVID-19 pandemic is going on - unless the patient is tested negative (ANSES, 2020).

##### COVID-19 Interactions With Herbs Modulating Host Cell Proteins

Coronaviruses have spike or surface (S) glycoproteins giving them their characteristic shape and name. These help the virus to enter the host cell by a receptor-mediated process (Lu et al., 2020). Although they show a complex pattern for receptor recognition, Angiotensin-converting enzyme 2 (ACE2) is considered the main host cell receptor of human pathogenic coronaviruses. In principle, herbal products down-regulating ACE2 may provide a baseline defense against infection. In the wake of SARS, Taiwanese researchers described emodin as an inhibitor of both S and ACE2 proteins. Emodin is present in medicinal plants such as *Rheum officinale* Baill. and *Polygonum multiflorum* Thunb (Ho et al., 2007).

However, some other plant metabolites have been identified as potential enhancers of ACE2 expression, including baicalin, tanshinones, magnolol, curcumin, and rosmarinic acid (ANSES, 2020). Therefore, French authorities have warned that the use of *Scutellaria* sp., *Magnolia* sp., *Salvia* sp., *Curcuma* sp., and *Rosmarinus officinalis* L., by COVID-19 patients, needs further evaluation (ANSES, 2020).

Immunomodulators may both stimulate or suppress the immune system by inhibiting and/or increasing the synthesis and release of pro-inflammatory mediators, including cytokines and eicosanoids. There is preclinical evidence of increased IL-1B and/or IL-18 production in infected immune cells by the use of *Sambucus nigra* L., polysaccharide-rich extracts from medicinal mushrooms, *Echinacea angustifolia* DC. and *Echinacea purpurea* L. (Moench.), Larch (*Larix* sp.) arabinogalactans-rich extracts, and plant extracts or food supplements rich in Vitamin D (Alschuler et al., 2020).

##### COVID-19 Interactions With Herbal Medicines Acting as Antithrombotic

The incidence of venous thromboembolism (VTE) in hospitalized patients with COVID-19 has been a controversial matter during the pandemic. Initial studies seemed to point towards patients the benefits of VTE prophylaxis in emergency care. Currently, there is insufficient data to recommend for or against the use of thrombolytics or increasing anti-coagulant doses in hospitalized COVID-19 patients. More epidemiologic studies with better control for underlying comorbidities, prophylactic anticoagulation, and COVID-19-related therapies are needed (NIH, 2020).

The current recommendation is that anticoagulants/antiplatelet therapy should not be initiated for the prevention of VTE in non-hospitalized patients with COVID-19 unless there are other indications. Similarly, modern phytotherapy should avoid recommending the use of plants with anticoagulant effects as prophylaxis of thromboembolic events triggered by COVID-19 disease. Some of the herbal medicines here reviewed are known to have anti-coagulant activity on top of their beneficial effects upon cold/flu symptoms such as garlic (*A. sativum* L.) and guaco (*M. glomerata* Spreng.). Although caution needs to prevail in assessing whether they are providing a benefit that outweighs the potential risks in case the disease gets complicated. This assessment needs to take into consideration quantitative scientific criteria that, in many times, are lacking. A case in point is the ‘zero tolerance’ to garlic, despite experimental evidence showing that an equivalent of 0.6% allicin does not impair platelet function and does no potentiate platelet-inhibiting drugs (Scharbert et al., 2007).

#### Herb-Drug Interactions in the Context of COVID-19 Emergency Treatment

Standard protocols in case of pneumonia contemplate assisted respiration for long periods, which in turn requires general anesthesia. Concerns about herb-drug interaction in anaesthesiology have been historically a major driver for objections to herbal medicine from clinicians. There is a substantial body of knowledge of how herbal medicines interact with anesthesia (Levy et al., 2017; AANA, 2020). There is a consensus among anesthesiologists in advising against any of the following herbal medicines that may be taken for flu symptoms:

- Echinacea (*Echinacea* sp.), because they may (theoretically) increase the risk of liver damage in patients under anesthesia.
- Ginkgo (*Ginkgo biloba* L., Ginkgoaceae), St. John’s wort (*Hypericum perforatum* L., Hypericaceae), and valerian (*Valeriana officinalis* L., Caprifoliaceae) because they may increase the effects of anesthesia and make it harder to wake up. They may also cause irregular heart rhythms.
- Ginseng (*Ginseng* sp., Araliaceae), licorice (*Glycyrrhiza glabra* L.), and milk thistle [*Silybum marianum* (L.) Gaertn.], because they may cause high blood pressure and a rapid heart rate.
- Garlic (*Allium sativum* L., Amaryllidaceae), ginkgo, green tea [*Camellia sinensis* (L.) Kuntz., Theaceae], feverfew (*Tanacetum parthenium* L., Asteraceae), ginger (*Zingiber officinale* L.) and Saw palmetto [*Serenoa repens* (W. Bartram) Small, Arecaceae], because they may cause prolonged bleeding.
- Garlic, in addition, can increase the effects of some OTC pain relievers.
- Ephedra: Several studies and clinical trials have been carried out to identify drugs that can effectively treat the disease, but, at the moment, the strategies to deal with the infection are only supportive (Cascella et al., 2020). Two recent reviews to on Chinese Herbal Medicine (CHM) can illustrate this. In the first one, the authors presented some CHM formulae used in the H1N1 outbreak that can be useful to prevent COVID-19 (Luo et al., 2020). The other offers guidelines for the treatment of COVID-19, at different stages, using CHM (Ang et al., 2020a). Among the most cited plant drug, *Ephedra* plays an important role. Both indicate a lack of evidence for the efficacy of those CHM preparations. The presence of a few medicinal plants such as *Ephedra* in the suggested guidance for COVID-19 is a subject of major concerns. Although *Ephedra*, listed in the WHO monographs (WHO, 1999), has been used for the treatment of asthma, cough, cold as well as in the management of weight loss (its primary use as a supplement in the USA), globally regulatory agencies have banned food supplements and medicines containing the ephedra alkaloid ephedrine, due to the serious adverse effects especially on the cardiovascular and nervous system and reported deaths (FDA, 2008; EFSA, 2013; EMA, 2015b).

### Benefits/Risks Assessment of Herbal Medicines Officially Referred as to as Useful to Relieve Symptoms Related to Respiratory Conditions

There have been a few metanalyses of the impact of herbal medicines in the treatment of common flu (Wagner L. et al., 2015; Ang et al., 2020b). Here, the analysis of the level of evidence was done considering COVID-19 and the current knowledge about symptoms and mechanisms of action involved in the coronavirus infection. In order to support the decision, the same criteria used for three drugs currently used to mitigate the same symptoms defined here: fever (paracetamol), inflammation (ibuprofen), and cough (codeine) were applied to the selected herbal medicines.

A modified PrOACT-URL approach has been used here as a tool for the evaluation of herbal medicines. Based on the defined criterion and the benefits/risks assessment, thirty-six herbal medicines, and three conventional drugs were evaluated (**Table 4**).

**Table 4:** Benefits/risks assessment in adult patients without any other conditions suffering early/mild flu symptoms in the context of COVID-19.

Treatment		Evidence Levels in Respiratory conditions^1^							Overall Benefit	Adverse Effects^2^	Overall Safety	Benefits/Risks
Plant Species/Drug	Pharmacopoeial name	Cold	Flu	Bronchitis	Asthma	Cough	Pain	Fever				
*Allium sativum*/bulbs, powder	Allii sativi bulbus (EMA)	Ib							High	B-II	Medium^a^	Promising
*Althaea officinalis/*roots, leaves	Althaeae radix					Ib			High	A-IV	High	Positive
*Andrographis paniculata/*leaves	Andrographidis paniculatae folium	Ib				Ib	Ib		High	D-III	Medium	Promising
*Commiphora molmol/*gum	Myrrha gummi-resina						1b		High	A-Ic	High	Positive
*Cymbopogon citratus/*leaves	Cymbopogonis folium	IV	IV						Low	A-IV	High	Unknown
*Echinacea angustifolia/*roots	Echinaceae angustifoliae radix	Ib	Ib						High	D-IV	Medium	Promising
*Echinacea pallida/*roots	Echinaceae pallidae radix	IV	IV						Low	D-IV	Medium	Unknown
*Echinacea purpurea/*herb	Echinaceae purpureae herba	Ib	Ib						High	D-IV	Medium	Promising
*Eucalyptus globulus/*essential oil	Eucalypti aetheroleum			III		III			Medium	A-IV	High	Promising
*Eucalyptus globulus/*leaves	Eucalypti folium			IV		IV			Low	A-IV	High	Unknown
*Foeniculum vulgare/*fruits	Foeniculi amari/dulcis fructus	IV				IV		IV	Low	A-IV	High	Unknown
*Glycyrrhiza glabra/*roots	Liquiritiae radix				Ib				High	A-II	High	Positive
*Hedera helix/*leaves	Hederae helicis folium			Ib	Ib	Ib			High	A-Ic	High	Positive
*Justicia pectoralis/*leaves	*Justicia pectoralis* folium					Ib			High	B-IV	Medium	Promising
*Magnolia officinalis*/bark	Magnoliae cortex				III				Medium	C-III	Medium	Promising
*Malva sylvestris/*leaves	Malvae sylvestris folium					IV			Low	A-IV	High	Unknown
*Mikania glomerata/*leaves	*Mikania glomerata* folium				Ib	IV			High	C-IV	Medium	Promising
*Ocimum gratissimum/*leaves	Ocimi Sancti folium	IV	IV	IV	IV			IV	Low	A-IV	High	Unknown
*Pelargonium sidoides/*roots	Pelargonii radix	Ia		Ib		Ib			High	D-IV	Medium	Promising
*Pimpinella anisum/*fruits essential oil	Anisi aetheroleum/fructus				Ib	IV		IV	High	C-IV	Medium	Promising
*Plantago lanceolata/*leaves	Plantaginis lanceolatae folium			IV		IV		IV	Low	A-IV	High	Unknown
*Platycodon chinensis/*roots	Platicodi radix					IV			Low	A-III	High	Unknown
*Polygala senega*/roots	Polygalae radix			IV		IV			Low	A-IV	High	Unknown
*Polypodium vulgare/*rhizome	Polypodii rhizoma					IV			Low	A-IV	High	Unknown
*Potentilla erecta/*rhizome	Tormentillae rhizoma					IV			Low	A-III	Medium	Unknown
*Primula veris/*roots	Primulae radix					IV			Low	B-III	High	Unknown
*Salix* sp./bark	Salicis cortex	IV	IV				Ib	IV	High	C-IV	Medium	Promising
*Sambucus nigra/*fruits	Sambuci fructus	Ia	Ia						High	A-Ic	High	Positive
*Scutellaria baicalensis/*roots	Radix Scutellariae			IV				IV	Low	C-III	Medium	Unknown
*Silybum marianum/*fruits	Silybi mariani fructus							IV	Low	A-Ic^b^	High	Unknown
*Thymus vulgaris/*herb essential oil	Thymi herba/aetheroleum					IV			Low	A-IV	High	Unknown
*Tilia cordata/*flowers	Tiliae flos	IV							Low	A-IV	High	Unknown
*Zingiber officinale/*rhizome	Zingiberis rhizoma	IV	IIa^*^		IV	IV			Medium	B-II	Medium	Promising
Ibuprofen	–	Ia	IV				Ia		Medium	B-II	Medium	Promising
Codeine	–					IV			Low	E-Ib	Low	Negative
Paracetamol	–	IV	IV						Low	E-Ib	Low	Negative

(1) Grading as per **Table 1**;(2) Codes and Grading as per **Table 2**;(3) As per **Table 3**; (a) Products with less than 0.6% allicin content are safe (Scharbert et al., 2007); (b) Chronic administration of products with 7%–8% silymarin content are safe up to 40 months (Corchete, 2008); (*) Clinical experiment in SARS patients (Vahdat Shariatpanahi et al., 2013).

The potential usefulness of the herbal medicines in the management of early symptoms of flu in the context of the current COVID-19 pandemic was compared with the baseline benefits/risks assessment performed on the reference drugs paracetamol (acetaminophen), ibuprofen and codeine. These drugs resulted not to be backed up by robust clinical evidence, and their safety profile was concerning in some cases. According to this ‘baseline’, five herbal medicines were found as potentially valid candidates in managing early or mild symptoms of cold, flu and bronchitis in the context of COVID-19 as they provide with ample safety margins and good evidence for efficacy: *Althaea officinalis*, *Commiphora molmol, Glycyrrhiza glabra, Hedera helix*, and *Sambucus nigra*. The authors recommend starting their integration into clinical advice as adjuvant therapies for respiratory diseases, even in the context of COVID-19.

A second group of twelve herbal medicines is to be considered as promising candidates, due to their reasonable safety margins and emerging evidence for efficacy: *Allium sativum*, *Andrographis paniculata*, *Echinacea angustifolia, Echinacea purpurea, Eucalyptus globulus* essential oil*, Justicia pectoralis, Magnolia officinalis, Mikania* glomerata *Pelargonium sidoides*, *Pimpinella anisum*, *Salix* sp, and *Zingiber officinale*. The authors recommend the scientific community to prioritize working on these herbals towards their full integration into clinical use. These two groups are, in the opinion of the authors, comparable in terms of safety -and sometimes efficacy- with the three reference drugs currently in clinical use for the target symptoms, so these herbal medicines may help to mitigate the discomfort in the early stages of the disease, based on their anti-inflammatory, immunomodulatory, and antitussive properties.

The following plants have good evidence for the treatment of asthma: *Glycyrrhiza glabra*, *Magnolia officinalis*, *Mikania glomerata*, and *Pimpinella anisum*. These plants cannot be candidates to treat respiratory infections but may be safe to use if the asthmatic patient suffers flu symptoms.

The rest of herbal medicines (approximately half of the analyzed herbal medicines) appear to have a good safety profile, but simply there is not enough evidence – clinical or pre-clinical – about their action on the target symptoms (cough, fever) or conditions (cold, flu). Therefore, they must be categorized as of ‘unknown’ potential and would need much more research to build up the necessary evidence to justify their use in the management of flu, let alone in the context of COVID-19.

### Benefits/Risks Assessment of Herbal Remedies Not Covered by International Monographs

Several food/herbal-based remedies are associated with the treatment of common cold and flu because they include vitamins (such as lemon juice), aromatic principles with perceived benefits for the upper respiratory system (such as mint, bayleaf), or because they are culturally considered as a “cure-all” (such as Nigella). Some of them are presented to the public in a “pharmaceutical forms” (capsules, tablets, syrups), thus looking like a “safe and effective medicinal approach”. The regulatory status of these products varies from country to country, and we advise here that only products that are regulated as (herbal) medicines should be used since this is an essential requirement for a product’s safety.

The following herbals and foods were identified by the authors as falling into this category: *Citrus limon, Culcitium canescens, Laurus nobilis, Lippia graveolens*, and *Plectranthus amboinicus*. We subjected the evidence for the efficacy and safety of these plants to the same Benefit/Risks assessment applied to the “officially recognized” herbal medicines, and the collected preclinical, clinical, and safety data are provided.

Nigella may be considered as a borderline “promising” treatment example as there is some preliminary clinical evidence for its relief of cough but it seems to be restricted to asthma patients, so from a physiopathological point of view is not clear how useful it would be in cold/flu. As for the others, although considered safe, there is simply not enough evidence – clinical or pre-clinical – in relation to their benefits on the target symptoms (cough, fever) or conditions (cold, flu).

Despite their lack of evidence in the treatment of flu, lemon, bay leaves, mint, and Nigella are deeply ingrained ‘polyvalent’ remedies in many cultures all over the World. Therefore, a succinct discussion on their efficacy and safety in the treatment of respiratory conditions is essential:

- The juice or tea of *Citrus limon* (L.) Burm.f fruits, alone or associated with salt, honey or ginger has been used to relieve cough and fever related to flu and cold in several traditional systems (Papp et al., 2011; Panizza et al., 2012; Sultana et al., 2016; Klimek-Szczykutowicz et al., 2020). However, there is no clinical evidence of such action in the respiratory tract, although this species is well-known as a rich source of vitamin C (Ye, 2017).
- Besides the use as food, the decoction of *Laurus nobilis* L. dried leaves is taken orally to treat respiratory distress (Chevallier, 1996; Ross, 2001), and the only information on *L. nobilis* posology is given by the American Pharmaceutical Association (Duke et al., 2002). The closest to an applicable study is an *in vitro* study examining SARS-CoV and the effect of several essential oils (Chen et al., 1995). The essential oil of *L. nobilis* leaves was also evaluated for its antinociceptive and anti-inﬂammatory activities in mice and rats (Sayyah et al., 2003). However, the lack of clinical evidence led to concerns about the use of this species.
- *Mentha* x *piperita* L. (peppermint) is widely used to treat bronchitis, fever and other respiratory disorders (WHO, 2002; Blumenthal, 2003), although no evidence can be found to corroborate these actions. The essential oil contains, among other compounds, menthol, menthone, menthofuran, and pulegone, a well-known hepatotoxin (Engel, 2003), leading to a deep concern about the use of essential oil (EMA, 2016e). However, peppermint tea usually contains a small amount of these compounds, and the normal use rarely led to serious adverse effects (EMA, 2008a).
- *Nigella sativa* L. (black seeds) is globally known as a spice and as a food item. In Arabic medicine, this species has very high status as a herbal medicine used for a wide range of diseases. In the context of COVID-19, the relevant uses are for asthma and in the more general management of inflammatory conditions. With regards to respiratory diseases its use can be traced back at least to Avicenna (ca. 980 – 1037, C.E.), who indicated it for shortness of breath and for stopping phlegm and Imam Ibn Qayyim Al-Jawziyya (1292–1350 C.E.), who recommended it for gasping and hard breathing (Koshak et al., 2018). In many Arabic Countries, “black seed” is used for asthma and cough, among many other uses, especially of gastrointestinal diseases (like abdominal pain, stomach ache, colic), rheumatism, skin diseases and recently use to manage diabetes have become popular (Aisa et al., 2019). Some small studies demonstrate clinical benefits in asthma patients (Koshak et al., 2017). Although black seed may be useful in the symptomatic relief of respiratory symptoms, especially associated with the severe asthmatic cough, the clinical evidence is very limited. A particular concern, in this case, are the many well-intended but very low-quality studies and the broad range of claims they try to support, making any assessment problematic.

### Limitations of the Present Work

The authors are aware of the limitations of this assessment: first, the body of studies is not large, even for reference drugs, and this is further complicated by the uncharted territory, which is the current pandemic. Second, the decision-making framework is here applied to herbal medicines for the first time and would need external validation.

The PrOACT-URL method is a valid approach to inform decision-making at high regulatory levels. The way we apply it here may well attract the critique of experts in the field of evaluation of the benefits/risks balance for medicines. We welcome constructive comments since this is exactly what we pursue: to start a high-level discussion on the potential of herbal medicines for the management of flu and their safety implications during this pandemic. For this, we need to work towards further adapting decision-making frameworks to bring about advances in ‘clinical phytotherapy’.

We acknowledge that the selection of herbal medicines is geographically limited and that many other species could have been considered. We took a “legalist” approach by only accepting entries provided by EMA or WHO, and we recognize we hold a collective expertise, especially in European and American herbal medicines. Nevertheless, the list includes globally-recognized, therapeutically relevant herbs. At the same time, there is, clearly, an emerging body of evidence on specific Asian (often multiherbal) preparations (especially TCM), which also is included in this Research Topic of Frontiers in Pharmacology, Sect. Ethnopharmacology, and thus these studies mutually complement each other.

## Conclusions

A total of 39 herbal medicines were identified as very likely to appeal to COVID-19 patients. According to our method, the benefits/risks assessment of the herbal medicines was found *positive* in 5 cases, *promising* in 10 cases, and *unknown* for the rest. On the same basis, only ibuprofen resulted promising, but we could not find compelling clinical evidence for a positive assessment to endorse the adjuvant use of paracetamol and/or codeine due to their common adverse effects in the respiratory function.

Our work suggests that *Althaea officinalis*, *Commiphora molmol, Glycyrrhiza glabra, Hedera helix*, and *Sambucus nigra* have safety margins superior to those of reference drugs and enough levels of evidence to merit their potential clinical use as adjuvants in the treatment of early/mild cases of COVID-19.

Herbal medicines are not a “magic bullet” to solve the problems related to flu - let alone COVID-19 or any other coronavirus - neither can avoid the virus infection but may alleviate symptoms and potentially improve the general wellbeing of patients. It requires a careful assessment of whether such adjuvant therapies are justified or not. Then again, it can only be a suitable therapy at a stage when the severity of the disease is minor. Overall, we highlighted the potential of some medicinal plants for the adjuvant management of early symptoms of flu within the context of COVID-19 in otherwise healthy adults. For an herbal medicine to be a medicine high quality, chemically well-characterized, and pharmacologically well-studied preparations are acceptable only. Equally, it will be essential that such well-characterized preparations are used in all future pharmacological and clinical studies (Heinrich et al., 2020).

In a broader context, this study also offers a novel approach for assessing the risks and benefits of using herbal medicines. Since the PrOACT-URL strategy is used here for the first time with a set of herbal medicines, we want to highlight the opportunities for a rigorous and evidence-based approach will have in a balanced assessment of such medicines.

## Author Contributions

DS Conceptualization of overarching research goals and aims, data/evidence collection, data curation, coordination of the overall work, formal analysis of study data, preparation, creation and/or presentation of the published work, critical review and revision. JP-G: Conceptualization of overarching research goals and aims, development and design of the methodology, visualization/data, formal analysis of study data, preparation, creation and/or presentation of the published work, critical review and revision. FB: Data/evidence collection, formal analysis of study data, critical review and revision. OE, YF-B, CJ, PM, EP, and MT: Data/evidence collection, formal analysis of study data. MH: Conceptualization of overarching research goals and aims, data/evidence collection, formal analysis of study data, critical review and revision.

## Funding

Coordenação de Aperfeiçoamento de Pessoal de Nível Superior, Capes-PrInt (88887468871/2019-00).

## Conflict of Interest

The authors declare that the research was conducted in the absence of any commercial or financial relationships that could be construed as a potential conflict of interest.
